# Repurposed Antipsychotics as Potential Anticancer Agents: Clozapine Efficacy and Dopaminergic Pathways in Neuroblastoma and Glioblastoma

**DOI:** 10.3390/life15071097

**Published:** 2025-07-12

**Authors:** Catarina Moura, Maria João Gouveia, Nuno Vale

**Affiliations:** 1 PerMed Research Group, RISE-Health, Faculty of Medicine, University of Porto, Alameda Professor Hernâni Monteiro, 4200-319 Porto, Portugal; cafsm13@gmail.com (C.M.); mariajoaogouveia@gmail.com (M.J.G.); 2 ICBAS—School of Medicine and Biomedical Sciences, University of Porto, Rua Jorge Viterbo Ferreira, 228, 4050-313 Porto, Portugal; 3 RISE-Health, Department of Community Medicine, Health Information and Decision (MEDCIDS), Faculty of Medicine, University of Porto, Rua Doutor Plácido da Costa, 4200-450 Porto, Portugal; 4 Department of Community Medicine, Information and Health Decision Sciences (MEDCIDS), Faculty of Medicine, University of Porto, Rua Doutor Plácido da Costa, 4200-450 Porto, Portugal

**Keywords:** dopamine, tyrosine, drug repurposing, neuroblastoma, glioblastoma, neuro-oncology, antipsychotic drugs, antiemetic drugs, H_2_O_2_, oxidative stress

## Abstract

Neuro-oncology focuses on the diagnosis and treatment of brain tumors, which, despite their rarity, are associated with high mortality due to their invasiveness and limited treatment options. Emerging evidence suggests that dopamine (DA), a neurotransmitter crucial for cognitive and emotional processes, and its receptors may influence tumor growth and the tumor microenvironment. This study aimed to evaluate the potential anticancer effects of repurposed antipsychotic dopamine-targeting drugs (Clozapine, CLZ; Pimozide, PIM; Olanzapine, OLZ; and Risperidone, RIS) and antiemetic drugs (Domperidone, DOM; Droperidol, DRO) on neuroblastoma (SH-SY5Y) and glioblastoma (A172) cell lines, and to assess whether their efficacy is modulated by oxidative stress and DA synthesis. The drugs were first tested individually, followed by co-treatment with tyrosine (Tyr), a dopamine precursor, and hydrogen peroxide (H_2_O_2_), an inducer of oxidative stress. Additionally, drug activity was evaluated in the simultaneous presence of H_2_O_2_ and Tyr. CLZ exhibited the highest cytotoxicity in both cell lines, suggesting strong anticancer potential and also synergism among the different combinations, particularly in SH-SY5Y. Liquid chromatography of the extracellular medium showed greater Tyr consumption in SH-SY5Y compared to A172 cells, indicating a higher dependence on extracellular Tyr to mitigate drug- and/or stress-induced cytotoxicity. In summary, several of the repurposed antipsychotics demonstrated cytotoxic effects on central nervous system tumor cells, with CLZ showing the most promising activity, even under oxidative stress conditions. These findings support further investigation into dopamine-targeting drugs as potential therapeutic agents in neuro-oncology.

## 1. Introduction

Neuro-oncology is a rapidly advancing field that integrates neurology and cancer biology, focusing on the diagnosis, treatment, and management of tumors affecting the central nervous system (CNS), including the brain and spinal cord, as well as neurological complications of cancer [1,2]. A key objective of neuro-oncology is to improve both the prognosis and quality of life for individuals with CNS tumors, such as glioblastoma and neuroblastoma. These malignancies pose significant challenges due to their aggressive nature and limited treatment options [3,4].

The World Health Organization classifies glioblastoma as a grade IV astrocytoma, making it the most common and aggressive primary malignant brain tumor, accounting for approximately 14.5% of all CNS tumors [5,6,7]. Originating from astrocytic glial cells, glioblastoma can occur at any age but is most frequently diagnosed around 65 years, with a higher prevalence in men than women [5,6]. Its incidence ranges from 3.19 to 4.17 cases per 100,000 person-years [5], establishing it as the most prevalent and aggressive primary brain tumor in adults [8]. Neuroblastoma, in contrast, is a pediatric malignancy that arises from embryonal tumors, specifically, from neural crest progenitor cells, and typically develops outside the cranial region [9]. As the most common extracranial solid tumor in children, neuroblastoma accounts for a significant proportion of pediatric cancers and contributes to approximately 15% of childhood cancer-related deaths [10,11]. With a median survival of 3 to 4 years, it ranks as the second most common pediatric malignancy, surpassed only by CNS tumors [11,12].

Recent advances in neuro-oncology have expanded the understanding of the molecular mechanisms underlying CNS tumors, including the role of neurotransmitter signaling in tumor progression. Among these, dopamine receptor (DR) signaling has emerged as a critical player in various cancers, including glioblastoma (GBM), and is now considered a relevant therapeutic target [13,14]. Studies indicate that DR expression is highly variable across different cancer types, with each tumor exhibiting a distinct DR expression profile. These alterations are observed in cancer cells and tumor-associated cells, suggesting a broader role for dopamine signaling in the tumor microenvironment [15]. Dopamine (DA), also known as 3,4-dihydroxytyramine, is a neurotransmitter primarily synthesized by dopaminergic neurons in the brain [16]. Its synthesis begins with the conversion of tyrosine (Tyr) into levodopa (L-DOPA) by tyrosine hydroxylase, followed by the decarboxylation of L-DOPA into DA. Once synthesized, DA is stored in synaptic vesicles and released into the synaptic cleft, where it binds to dopamine receptors (DRs) or undergoes reuptake for recycling [16,17]. Emerging evidence suggests that dopamine signaling in peripheral tissues is frequently dysregulated in cancer cells, revealing novel cancer-specific vulnerabilities. Notably, studies have shown that cancer patients receiving antipsychotic medications alongside standard anticancer therapy exhibited improved clinical outcomes, further highlighting the potential role of dopamine receptors (DRs) in tumor progression [16]. This growing body of research has fueled interest in repurposing dopaminergic ligands and neuropharmacological drugs for cancer therapy. Given the high costs, time-consuming nature, and frequent inefficacy of conventional drug development, leveraging existing dopaminergic drugs for oncological applications could provide significant benefits for both patients and drug developers [18].

**Figure 1:**
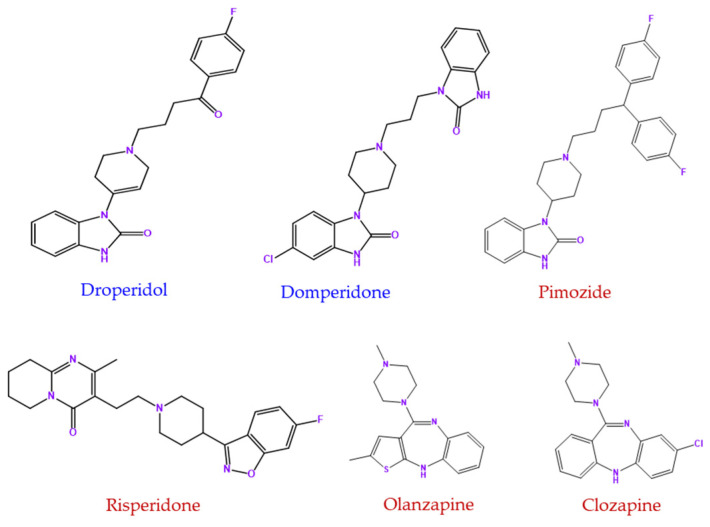
Chemical structures of the antiemetic (blue) and antipsychotic (red) drugs used in this study. The antiemetic drug droperidol is a butyrophenone, while domperidone belongs to the benzimidazole class. Among the antipsychotics, olanzapine and clozapine are benzodiazepines, risperidone is a benzisoxazole, and pimozide is classified as a diphenylmethane derivative (functional groups are highlighted in purple).

Motivated by these findings, this study explores the effects of six repurposed dopamine 2 receptor (D2R)-targeting drugs—two antiemetics and four antipsychotics—on glioblastoma and neuroblastoma cell lines. The antiemetics droperidol (DRO) and domperidone (DOM) act as D2 receptor antagonists (Figure 1). DRO, approved by the Food and Drug Administration (FDA) in 1970, has been used to manage psychosis, agitation, vertigo, and benign headaches, although it is primarily prescribed as an antiemetic in the United States [19,20]. Despite its effectiveness, the FDA issued a severe safety warning in 2001; yet, its potential anticancer properties remain largely unexplored. DOM, though widely used for nausea and vomiting in several countries, has never been FDA approved due to concerns about cardiac arrhythmias at high doses [21,22,23]. Unlike DRO, DOM primarily acts on CNS regions lacking a blood–brain barrier and is metabolized by CYP3A4, with some studies suggesting it may promote liver carcinoma cell proliferation, potentially counteracting dopamine’s tumor-suppressive effects [18,24]. The antipsychotics pimozide (PIM), risperidone (RIS), olanzapine (OLZ), and clozapine (CLZ) (Figure 1, red) were all FDA approved for treating psychiatric disorders and act by modulating dopaminergic and serotonergic signaling. PIM is a potent D2 receptor antagonist used to treat Tourette’s syndrome, chronic psychosis, and resistant motor and phonic tics, affecting multiple neurotransmitter pathways [25,26]. RIS, a second-generation antipsychotic, strongly antagonizes both D2 and serotonin 5-HT2 receptors, with a higher affinity for 5-HT2A [27,28,29]. OLZ, widely used for schizophrenia, bipolar disorder, and manic episodes, exhibits a high affinity for serotonin receptors (5-HT2A/2C, 5-HT3, 5-HT6, and 5-HT7) compared to dopaminergic receptors (D1–D5) [30,31]. Due to its affinity for several central nervous system receptors, OLZ also possesses antiemetic properties [30]. CLZ, considered the most effective antipsychotic for treatment-resistant schizophrenia, has a unique profile with a high affinity for D4 receptors and a lower affinity for D2 receptors [32,33,34]. However, its use is limited due to severe side effects and the need for frequent monitoring [32].

Although originally developed for the treatment of psychiatric disorders, accumulating evidence suggests that these drugs may also exhibit anticancer properties through various mechanisms. PIM has demonstrated antiproliferative effects in several tumor cell lines, targeting key pathways such as STAT5 phosphorylation, cell cycle arrest, and ERK signaling [25,26,35]. RIS has shown cytotoxic activity against proliferating gastric and colorectal cancer cells, in addition to exhibiting both cytotoxicity and proliferative effects in neuroblastoma cells (SK-N-SH) [36,37,38]. OLZ, beyond its role in managing cancer-related symptoms [31], has been reported to enhance the efficacy of chemotherapeutic agents in vitro [16] and to inhibit glioma cell growth when combined with temozolomide [39]. CLZ has also demonstrated potential anticancer activity by reducing acute myeloid leukemia cell numbers, though its precise mechanism of action remains unclear [40].

**Figure 2:**
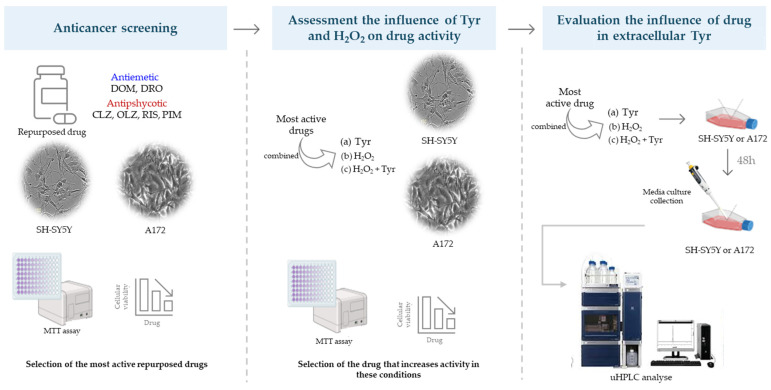
Schematic overview of the experimental workflow. SH-SY5Y (neuroblastoma) and A172 (glioblastoma) cell lines were used to evaluate the anticancer potential of six repurposed dopaminergic drugs. (1) Drug cytotoxicity was first screened via MTT assay. (2) The most active compounds were then tested in combination with L-tyrosine (Tyr), hydrogen peroxide (H_2_O_2_), or both, to assess the influence of amino acid availability and oxidative stress. (3) Finally, extracellular Tyr levels were quantified by uHPLC following 48 h exposure to selected drug combinations.

Given the urgent need for novel therapeutic strategies in neuro-oncology, this study aims to assess whether dopaminergic drugs could be effective in treating nervous system cancers that express dopamine receptors (DRs). To investigate their anticancer potential, we evaluated the effects of six repurposed dopaminergic drugs on human glioblastoma (A172) and neuroblastoma (SH-SY5Y) cell lines, both of which exhibit DR expression (Figure 2).

Since dopamine synthesis and signaling can be influenced by metabolic and oxidative factors, we also examined the impact of tyrosine (Tyr), a key precursor in dopamine biosynthesis, and hydrogen peroxide (H_2_O_2_), a reactive oxygen species involved in oxidative stress and cancer progression [18]. Tyrosine availability can modulate dopamine levels and receptor activation, potentially enhancing the therapeutic effects of dopaminergic drugs. Oxidative stress has been implicated in both tumor development and cell death pathways [18]. By incorporating these factors, this study provides a more comprehensive analysis of whether dopaminergic modulation influences cancer cell viability and extracellular dopamine levels, offering insights into their potential repurposing as oncological treatments.

## 2. Materials and Methods

### 2.1. Materials

Dulbecco’s modified Eagle’s medium (DMEM), fetal bovine serum (FBS), and a penicillin–streptomycin mixture (1000 U/mL; 10 mg/mL) were sourced from PAN-Biotech (Aidenbach, Germany). Thiazolyl blue tetrazolium bromide (MTT; cat. no. M5655), tyrosine (cat. no. T3754), and hydrogen peroxide (30%; PerhydrolTM; cat. no. 1.07209) were obtained from Sigma-Aldrich (Merck KGaA, Darmstadt, Germany). The six repurposed drugs, including CLZ (cat. no. C6305), DOM (cat. no. D122), DRO (cat. no. D1414), OLZ (cat. no. O1141), PIM (cat. no. P1793), and RIS (cat. no. R3030), were also obtained from Sigma-Aldrich.

### 2.2. Cell Culture

The study was performed in human neuroblastoma (SH-SY5Y) and glioblastoma (A172) cell lines obtained from American Type Culture Collection (ATCC, Manassas, VA, USA). The cells were cultured in Dulbecco’s modified Eagle’s medium (DMEM), supplemented with 10% fetal bovine serum (FBS) and 1% penicillin/streptomycin mixture and maintained at 37 °C in a humidified atmosphere with 95% air and 5% CO_2_. For maintenance, cells were cultured in a monolayer and sub-cultured by trypsinization in the same medium when the cells reached a confluence of approximately 80%. The culture medium was changed every 3–4 days. Before each experiment, trypsin (0.25% trypsin-EDTA) was used to detach the cells. Afterward, cells were centrifuged at 1100 rpm for a duration of 5 min utilizing a Hettich centrifuge (Tuttlingen, Germany) and then seeded at a density of 4.2 × 10^4^ cells/cm^2^ in 96-well plates for the viability assays.

### 2.3. Cell Treatment

CLZ, DOM, DRO, OLZ, PIM, and RIS were dissolved in 100% dimethyl sulfoxide (DMSO). Then, stock solutions at a concentration of 200 mM for olanzapine and risperidone and a concentration of 100 mM for the remaining drugs were prepared. After that, the stock solutions were diluted in culture media in a range of 0.01 to 100 µM with 0.1% DMSO. For the combinations, tyrosine (Tyr) and hydrogen peroxide (H_2_O_2_) were dissolved in sterilized water (0.1% in cell culture minimum), and stock solutions of 1000 mM for Tyr and 2 M for H_2_O_2_ were prepared. The amino acid Tyr was tested at concentrations of 25, 100, 250, and 500 µM in the cell lines, while H_2_O_2_ was only tested at a concentration of 132 µM (IC_50_ value determined by the research group at 48 h).

For compounds alone, the controls were culture media with 0.1% DMSO in cell culture media, while for combinations of the repurposed drugs with Tyr or H_2_O_2_, the controls were composed of 0.2% DMSO in a cell culture medium. Each treatment underwent testing over 24, 48, and 72 h following cell attachment to the plates.

The experimental workflow began with the assessment of the cytotoxic effects of individual compounds (drugs, Tyr, and H_2_O_2_). Based on these results, the most cytotoxic drugs were subsequently tested in combination with either Tyr or H_2_O_2_. Finally, the impact of the concurrent presence of Tyr and H_2_O_2_ on the anticancer activity of the selected drug was investigated.

### 2.4. Cell Viability Assay

Cellular viability was assessed at 24, 48, and 72 h following cell treatments using the MTT assay. After the removal of the culture medium, 100 µL of MTT solution (0.5 mg/mL in PBS) was introduced into each well. Then, protected from the light, the cells were incubated at 37 °C for 2 h. Subsequently, MTT was removed, and 100 µL of DMSO was added to each well. Finally, absorbance values at 570 nm were measured in the automated microplate reader (Tecan Infinite M200, Zurich, Switzerland) to evaluate the effects of the drugs alone and in combination on the cell viability of SH-SY5Y and A172 cells.

### 2.5. Cell Morphology Assessments

After administering the drug treatment, the morphological features of SH-SY5Y and A172 cells were examined and recorded utilizing a Leica DMI 6000 B Automated Microscope coupled to a Leica DFC350 FX camera (Leica Microsystems, Wetzlar, Germany). Cell images were analyzed on a computer through the application of Leica Las X imaging software (v3.7.4) (Leica Microsystems, Wetzlar, Germany).

### 2.6. Synergism Studies

The combination index (CI) of combinations was calculated according to the Chou–Talalay method using CompuSyn software (version 1.0; ComboSyn, Paramus, NJ, USA). CompuSyn software (version 1.0; ComboSyn, Paramus, NJ, USA) and the Chou–Talalay equation were used to assess the combination index (CI) of several drug combinations using a non-fixed ratio. In this context, a CI lower than 1 indicates synergism between the drugs, a CI equal to 1 indicates additivity, and a CI greater than 1 indicates antagonism. The Chou–Talalay method is based on the median effect equation derived from the law of mass action and integrates the Michaelis–Menten, Hill, Henderson–Hasselbalch, and Scatchard equations in biochemistry and biophysics [41].

### 2.7. Ultra-High-Performance Liquid Chromatography (uHPLC) Analysis

Medium cultures of treated cells with drugs, Tyr, and/or H_2_O_2_ were subjected to uHPLC to quantify the presence of Tyr or Dopamine in the extracellular medium. The separation was carried out at a flow of 2 mL/min (water/acetonitrile). The optical density of all the tested compounds was recorded at 280 nm. Quantification was performed based on standard 7 curves for L-Tyr and Dopamine. Results were analyzed using Chromera^®^ software, version 3.2.0, Perkin Elmer (Waltham, MA, USA).

Tyr concentrations between 0.01 and 1000 µM were used to generate the calibration curve. The resulting equation (y = 293.02 x + 2141.5; R^2^ = 0.9995) was used to calculate the Tyr concentration present in each analyzed sample, following the recommendations of the manufacturer’s manual.

### 2.8. Data and Statistical Analysis

GraphPad Prism 8 (GraphPad Software Inc., San Diego, CA, USA) was used to create bar graphs of the cell viability and to produce concentration–response curves by nonlinear regression analysis. The viability of cells treated with each drug was normalized to the viability of control cells, and cell viability fractions were plotted versus drug concentrations on a logarithmic scale.

Statistical analysis was performed in all experiments. The results are expressed as the arithmetic mean ± standard error of the mean (SEM) from three independent cell culture experiments. Differences between the treated cells and the corresponding untreated control were assessed using two-way ANOVA, followed by multiple comparisons with Dunnett’s post hoc test to compare against the controls or Tukey’s post hoc test to compare within treatment groups (multiple comparisons). The differences were considered statistically significant when the *p*-value < 0.05. All statistical analyses, graph constructions, and calculations of IC_50_ values were executed using GraphPad Prism 8 software.

## 3. Results

### 3.1. Repurposed Dopaminergic Drugs Have Cytotoxic Effects on SH-SY5Y and A172 Cell Lines

**Figure 3:**
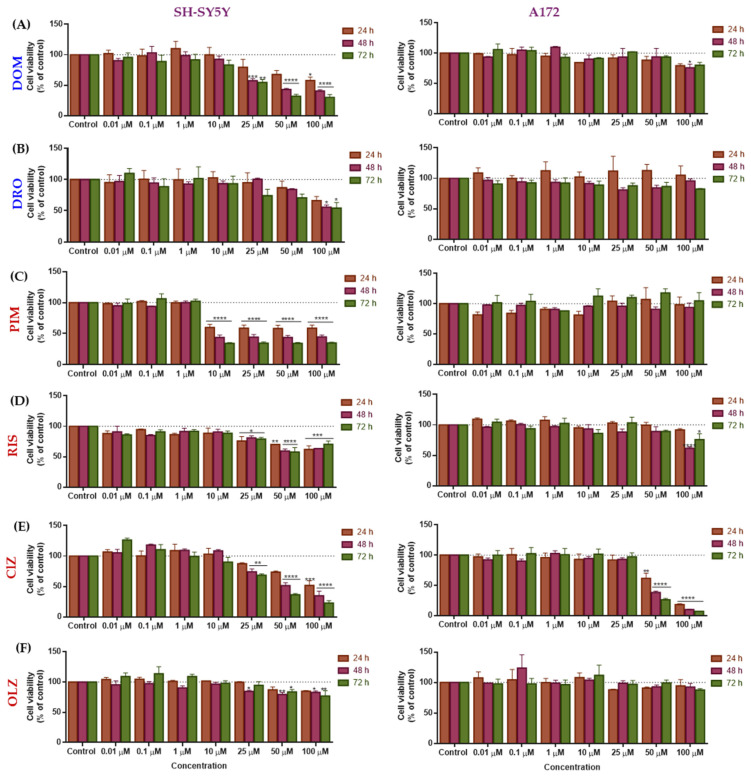
Effects of repurposed dopaminergic drugs antiemetic (blue) [DOM (**A**) and DRO (**B**)], and antipsychotic (red) [PIM (**C**), RIS (**D**), CLZ (**E**), and OLZ (**F**)] on the viability of SH-SY5Y and A172 cells. The cells were cultured in the presence of increasing concentrations of the six drugs and treated for 24, 48, and 72 h. Cell viability was determined using the MTT assay. Results are expressed as a percentage of the vehicle-treated control ± SEM of three separate experiments. Two-way ANOVA was used as a statistical test. Statistically significant * *p* < 0.05, ** *p* < 0.01, *** *p* < 0.001, and **** *p* < 0.0001 vs. control.

The repurposed drugs demonstrated potential anticancer activity in both cell lines (Figure 3 and Table 1). Morphological alterations of cells treated with these drugs are shown in Figures S1 and S2.

In the neuroblastoma (SH-SY5Y) cell line, antiemetic DOM and antipsychotics PIM and CLZ demonstrated statistically significant inhibitory effects on growth compared to the control (Figure 3A,C,E). The cytotoxic effect induced by DOM is concentration- and time-dependent, being more pronounced at a concentration equal to or above 25 µM and at 48 and 72 h (Figure 3A). The greatest decrease in the number of cells occurred at 72 h with the 100 µM concentration, where cell viability statistically significantly reduces to approximately 30%. The half-maximal inhibitory concentration (IC_50_) reduces approximately 2-fold from 24 h (IC_50_ = 30.3 µM) to 48 and 72 h (IC_50_ = 18.6 and 17.6 µM), suggesting that prolonged drug exposure increases cytotoxicity in this cell line (Table 1).

The cytotoxic effects of PIM were statistically significant compared with controls from a concentration equal to or above 10 µM, with cell viability of 34% at 72 h (Figure 3B). Interestingly, this effect remains steady at higher concentrations, i.e., at 72 h, the cell viability did not alter significantly with drug increase up to 10 µM. The lower IC_50_ was achieved at 48 h (IC_50_ = 1.8 µM) and the highest at 24 h (IC_50_ = 6.6 µM) (Table 1). CLZ exhibits a concentration- and time-dependent inhibition starting at 25 µM, with the lowest cell viability percentage reaching approximately 23% at a concentration of 100 µM after 72 h (Figure 3E). The IC_50_ value obtained for CLZ decreases through time, achieving the lowest value at 72 h (IC_50_ = 27.4 µM). Among the three most effective repurposed drugs, PIM demonstrated the greatest potential as an anticancer agent against neuroblastoma with the lowest IC_50_ value, followed by DOM and CLZ. The remaining drugs, DRO, OLZ, and RIS, showed no cytotoxicity and consequently no anticancer activity against neuroblastoma cells.

In A172 cells, DOM, DRO, PIM, and OLZ did not induce cytotoxicity effects (Figure 3 and Table 1). However, DRO and PIM exhibited neuroprotective effects (Figure 3B,C). DRO was demonstrated to have anticancer effects at 24 h, as evidenced by an increase in cell viability above 100%, particularly at the 50 µM concentration, which reached approximately 113% (Figure 3B). However, this increase was not statistically significant when compared to the control, except for the concentrations of 0.1 µM and 10 µM, where no significant increase in viability was observed. PIM demonstrated neuroprotective activity only at higher concentrations (>10 µM) and most significantly at 72 h. Similar to DRO, these increases were not statistically significant when compared to the control.

On the contrary, CLZ and RIS exhibited cytotoxic effects (Figure 3 and Table 1). This activity was demonstrated by CLZ at concentrations above 25 µM and is dependent on the time and concentration (Figure 3E). The lowest cell viability (~7%) was observed for the 100 µM concentration at 72 h in the glioblastoma cells treated with the highest concentration of CLZ. This highlights the potential anticancer activity of CLZ in this cell line. Interestingly, CLZ was cytotoxic in both cell lines, with activity 2-fold more pronounced in neuroblastoma (Table 1). RIS presented moderate cytotoxicity activity compared with CLZ (Figure 3D). Its activity is more significant at the highest concentrations (>25 µM). The IC_50_ of these drugs at 48 h demonstrated that CLZ (IC_50_ = 42.2) is more active than RIS (IC_50_ = 51.6 µM) (Table 1).

Overall, these findings suggest that CLZ is promising for treating glioblastoma and neuroblastoma diseases, while DOM and PIM may be more selective for neuroblastoma cells.

### 3.2. Evaluation of the Effect of the Presence of Dopamine Precursors and Stress Oxidative Inductors on the Cytotoxicity Effect of Repurposed Drugs on SH-SY5Y and A172 Cells

Initially, the effects of Tyr and H_2_O_2_ individually on cell viability were assessed in both cell lines. Subsequently, to investigate whether Tyr and the oxidative stress inducer H_2_O_2_ modulate the cytotoxicity of the repurposed drugs, cells were exposed to the drugs in combination with either Tyr or H_2_O_2_, followed by treatment with both agents concurrently.

#### 3.2.1. Tyrosine Did Not Induce Cytotoxicity in Cell Lines but Enhances the Cytotoxicity of CLZ on SH-SY5Y and A172 Cells

**Figure 4:**
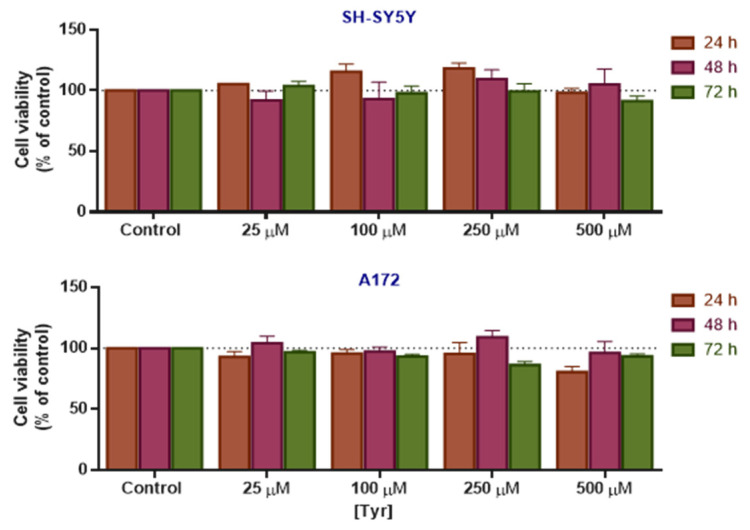
Effect of Tyr on the viability of SH-SY5Y and A172 cells. The cells were cultured in the presence of increasing concentrations of Tyr for 24, 48, and 72 h. Cell viability was determined using the MTT assay. Results are expressed as a percentage of the vehicle-treated control ± SEM of three separate experiments.

Firstly, SH-SY5Y and A172 cell lines were treated with increasing concentrations of Tyr, and cell viability was evaluated using the MTT assay (Figure 4). No statistically significant differences in cell viability were observed in comparison to untreated controls, indicating that Tyr did not exert cytotoxic effects under the tested conditions.

This lack of toxicity is further supported by the cell morphology, which showed no changes in the shape or size of the cells in either cell line (Figure S3). Interestingly, in SH-SY5Y at 100 and 250 µM at 24 h of exposure, Tyr induced a moderate anticancer effect since the cell viability was higher than 100% in comparison with controls, yet it is not statistically significant (Figure 4). This effect is less pronounced in A172 cells. By contrast, at the highest concentration, Tyr is slightly cytotoxic, reducing cell viability in a non-statistically significant manner at 24 h in SH-SY5Y and A172.

**Figure 5:**
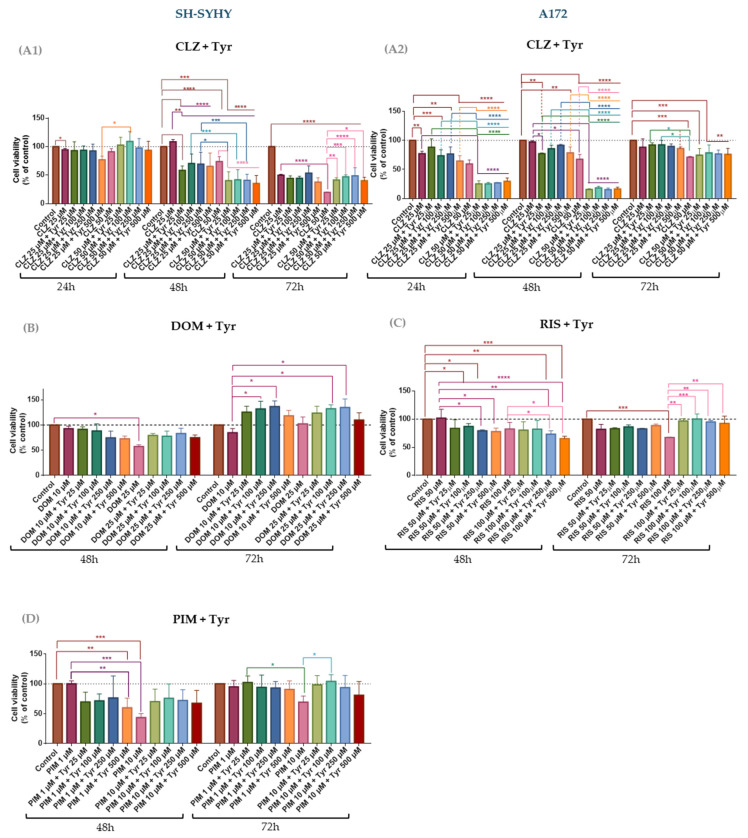
Effect of the most promising anticancer agents, CLZ (**A1**), DOM (**B**), and PIM (**C**), combined with Tyr on the viability of SH-SY5Y cells and CLZ (**A2**) and RIS (**D**) combined with Tyr on A172 cells. The cells were cultured in the presence of two concentrations of CLZ, DOM, and PIM and with increasing concentrations of Tyr and treated for 24, 48, and 72 h. Cell viability was determined using the MTT assay. Data are expressed as a percentage of the vehicle-treated control mean ± SEM of three separate experiments. Statistically significant * *p* < 0.05, ** *p* < 0.01, *** *p* < 0.001, and **** *p* < 0.0001 vs. indicated group.

Based on the results described in Section 3.1, the most promising repurposed drugs were selected to assess how the presence of Tyr influences their activity on SH-SY5Y and A172 cell lines. Thus, CLZ, DOM, and PIM were evaluated in combination with Tyr on SH-SY5Y cells. Similarly, CLZ and RIS were combined with Tyr on A172 cells. Figure 5 depicts the cell viability percentage obtained from the MTT assay, and cell morphological alterations are illustrated in Figures S4–S8.

The combinatorial effect of CLZ and Tyr exhibited distinct patterns in SH-SY5Y and A172 cells, highlighting cell-type-specific responses to the treatment (Figure 5). At 24 h, the co-treatment of SH-SY5Y with CLZ with Tyr increased cell viability compared with the drug alone, especially at higher concentrations (e.g., CLZ 50 µM), where cell viability is above 100%, which might suggest that Tyr might induce an anticancer effect (Figure 5A1). By contrast, at 48 h, the combinations of Tyr with either CLZ 25 µM or 50 µM reduce cell viability in a statistically significant manner in comparison with the drug alone (*p* < 0.0001). This is more evident in combination with the highest drug concentration (Figure 5A1). However, at 72 h, CLZ 25 µM induces similar cell viability as in combination (e.g., 49.9% CLZ 25 µM vs. 44.5% CLZ 25 µM + Tyr 100 µM), and at the highest concentration, the drug alone is more cytotoxic than when combined with Tyr (19.6% CLZ 50 µM vs. 41.5% CLZ 50 µM + Tyr 100 µM). The time of exposure and the Tyr concentration might influence CLZ activity on SH-SY5Y cells. These dynamic effects were further supported by combination index (CI) analysis using the Chou–Talalay method: synergism was observed at 72 h (CI range 0.3500–0.7630), whereas antagonism was predominant at earlier time points (CI > 1), reinforcing the notion that the timing of exposure critically influences the therapeutic outcome in SH-SY5Y cells (Table 2).

In contrast, the combination of CLZ and Tyr was more cytotoxic at 24 h and 48 h in A172 glioblastoma cells (Figure 5A2). At 24 h, low-dose CLZ (25 µM) in combination with increasing concentrations of Tyr did not significantly alter cell viability compared to CLZ alone. However, the combination of 50 µM CLZ with higher Tyr concentrations induced a marked reduction in cell viability at 24 and 48 h compared with the drug alone, suggesting a potentiation of CLZ’s cytotoxic effect. Notably, this enhancement was not sustained at 72 h, when cell viability returned to levels comparable to CLZ alone. The cell viability of combinations increased compared to those obtained at 48 h, especially in combinations with CLZ 50 µM (e.g., CLZ 50 µM + Tyr 250 µM at 48 h 15.2% vs. 76.5% at 72 h). CI analysis revealed a synergistic interaction at 24 h (CI range 0.2576–0.4137), whereas high antagonism was observed at 48 and 72 h (CI > 1) (Table 2), explained by the increase in cell viability through time exposure. The presence of Tyr and the drug elicit synergism in both cell lines at different time points. Nevertheless, this demonstrated a greater therapeutic potential of CLZ in cells that utilize Tyr as neurons.

In SH-SY5Y cells, combining DOM with varying concentrations of Tyr did not result in significant changes in cell viability when compared to DOM alone (Figure 5B). At 72 h, several combinations led to cell viability values exceeding 100%, suggesting a potential anticancer effect associated with Tyr. Consistent with these findings, the combination index (CI) values for these combinations were above 1, indicating an antagonistic interaction (Table 3). The sole exception was observed with the combination of DOM at 10 µM and Tyr at 25 µM, which yielded a CI value close to 1, suggesting an additive effect. Generally, the combination of PIM 1 µM with different Tyr concentrations resulted in a significant reduction in cell viability when compared to PIM alone, particularly at 48 h (e.g., 59.8% PIM 1 µM + Tyr 500 µM vs. 99.9% PIM 1 µM) (Figure 5C). This enhanced cytotoxicity was supported by CI values below 1, indicating a synergistic effect (Table 3). The combination of the highest concentration of PIM (10 µM) with increasing concentrations of Tyr increased cell viability relative to the drug alone, revealing an antagonism effect (Table 3). This trend was more pronounced at 72 h, with statistically significant differences in the cell viability of combinations compared with the drug alone (Figure 5C). These findings suggest that both concentrations of PIM and the duration of exposure critically influence the nature of its interaction with Tyr.

In A172 cells, combining RIS with varying concentrations of Tyr resulted in an increase in cell viability compared to RIS alone (Figure 5D). This effect was consistent in all tested conditions and suggests an antagonistic interaction between the two compounds. Supporting this observation, the combination index (CI) values for all RIS–Tyr treatments were greater than 1, indicating a high degree of antagonism (Table 3). These findings highlight the limited therapeutic potential of this combination in glioblastoma cells and further underscore the importance of evaluating drug interactions in a cell-type-specific manner.

In conclusion, CLZ in combination with Tyr remains cytotoxic against SH-SY5Y and A172 cells and demonstrated synergism, counteracting the potential anticancer effect of Tyr.

#### 3.2.2. Anticancer Activity of Repurposed Drugs Is Potentiated in the Presence of an Oxidative Stress Inducer in SH-SY5Y

**Figure 6:**
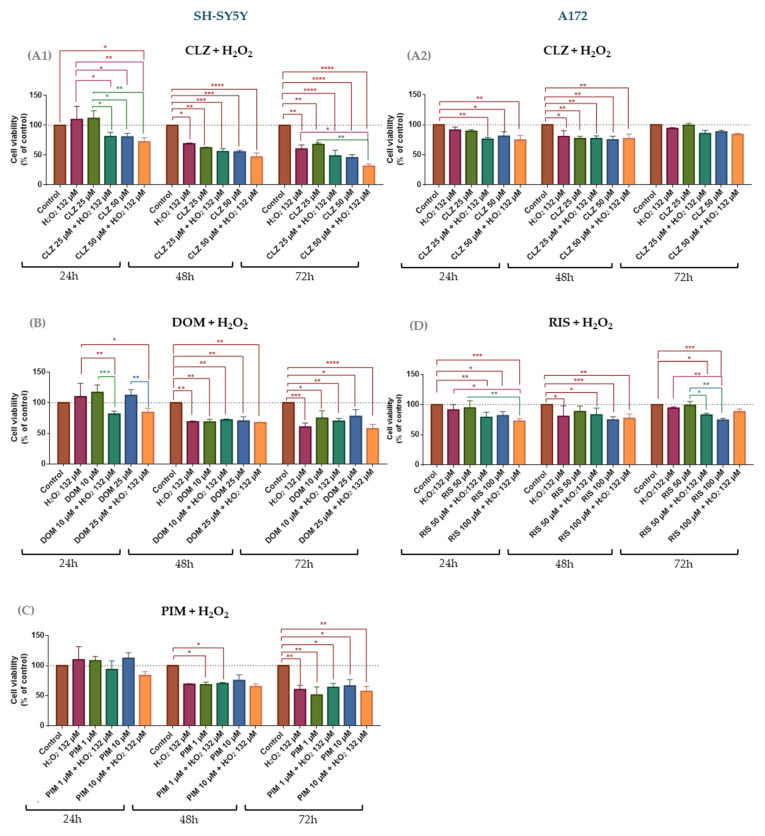
Effect of the presence of stress oxidative inducer (H_2_O_2_) on cytotoxicity of CLZ (**A1**), DOM (**B**), and PIM (**C**) on SH-SY5Y cells and CLZ (**A2**) and RIS (**D**) on A172 cells. The cells were cultured in the presence of two concentrations of CLZ, DOM, and PIM and with a unique concentration of H_2_O_2_ and treated for 24, 48, and 72 h. Then, cell viability was determined using the MTT assay. Results are expressed as a percentage of the control and represent means ± SEM of three separate experiments. Statistically significant * *p* < 0.05, ** *p* < 0.01, *** *p* < 0.001, and **** *p* < 0.0001 vs. identified group.

It has been suggested that H_2_O_2_ acts as an oxidative stress inducer that triggers responses in the cycle arrest, apoptosis, and modulation of genes related to oxidative stress in breast cancer tumor cells [16,40]. Herein, to assess whether oxidative stress modulates the cytotoxicity of repurposed drugs, SH-SY5Y and A172 cells were treated with H_2_O_2_ alone or combined with repurposed drugs. Viability measurements (Figure 6) and morphological analyses (Figures S9–S13) revealed that SH-SY5Y is more susceptible to oxidative stress than A172 cells, underscoring a cell-type-specific vulnerability to oxidative stress.

In SH-SY5Y, co-treatment with CLZ and H_2_O_2_ is time-dependent since the cell viability decreases through time, achieving a lower value at 72 h (Figure 6A1). Additionally, it is more significant in combination with the highest drug concentration, which suggests that it is concentration-dependent. Comparing the cell viability of CLZ 25 µM (67.8%) with the combination of CLZ 25 µM and H_2_O_2_ (48.7%), the addition of H_2_O_2_ resulted in a statistically significant decrease in cell viability compared with the control and drug alone, which is supported by the CI value that confirms the synergism between the compounds. Increasing the drug concentration reduces the cell viability compared with the controls; however, the differences between the drug alone and co-treatment are less pronounced and non-statistically significant (e.g., CLZ 50 µM 45.6% vs. CLZ 50 µM plus H_2_O_2_ 31.5% at 72 h). Although direct comparisons of the combination versus H_2_O_2_ or CLZ alone did not always reach statistical significance, in general, there is a cell viability reduction of combinations compared to the drug alone, which indicates a synergistic interaction (CI < 1) (Table 4).

At 24 h, the co-treatment of DOM and H_2_O_2_ (Figure 6B) statistically significantly decrease cell viability compared with the drug alone, especially when the drug was used at a lower concentration (e.g., 116% DOM 10 µM vs. 81.4% DOM 10 µM + H_2_O_2_ 132 µM). Despite at 48 h the cell viability of the drugs and combinations were lower than at 24 h, there is no significant difference between the drug alone and the combination (e.g., 68.5% vs. 72.0%). Curiously, at 72 h, the cell viability of the drugs in different concentrations were slightly higher than at 48 h (e.g., DOM 10 µM at 48 h 68.5% vs. 74.8% at 72 h), and the values for combinations were lower than those for the drug alone (e.g., 77.8% DOM 25 µM vs. 57.4%) but not statistically significant. At 48 and 72 h, the cell viability of H_2_O_2_ alone was similar or lower than the drug alone, which might explain the antagonism observed (CI > 1 at 48–72 h), while at 24 h synergy was indicated (CI < 1) (Table 4).

In general, the combination of PIM with H_2_O_2_ was more cytotoxic than the drug alone in a time- and concentration-dependent manner (Figure 6C). In general, the combination with PIM 10 µM reduced the cell viability more pronouncedly than PIM 1 µM (e.g., at 72 h, 57.2% vs. 63.8%). Interestingly, at 72 h, comparing PIM 1 µM plus H_2_O_2_ with the drug alone (1 µM), it was observed that the drug alone was more active in reducing cell viability alone than in combination (51% vs. 64%), whereas at the higher dose (10 µM), the addition of H_2_O_2_ further decreased viability relative to the drug alone (57.2% vs. 67.1%) (Figure 6C). Although direct comparison between the combination versus the drug alone was not statistically significant, the CI demonstrated that most combinations present synergism or additivity (CI <= 1), except for the lowest concentration of PIM at 24 h (Table 4).

In A172 glioblastoma cells, co-treatment with CLZ and H_2_O_2_ did not markedly enhance cytotoxicity (Figure 6A2), as it did in SH-SY5Y cells. At 24 and 48 h, there is a slight decrease in the cell viability of combinations in comparison with the drug alone, but in a non-statistically significant manner. At these points, particularly at the lower CLZ concentration, Chou–Talalay analysis revealed synergistic interactions (CI < 1) (Table 5). Interestingly, extended exposure (72 h) of cells to different compounds increased cell viability and toward antagonism (CI > 1), thereby blunting CLZ’s efficacy (Table 5). Although both 25 µM and 50 µM CLZ plus H_2_O_2_ produced statistically significant viability reductions versus controls, overall cell death remained modest: the greatest effects occurred at 24 h, with ~24% cell death (76% viability) for 25 µM CLZ + H_2_O_2_ and ~26% cell death (74% viability) for 50 µM CLZ + H_2_O_2_.

Similarly, the combination of RIS and H_2_O_2_ elicited only modest cytotoxicity (Figure 6D). At 24 h, there are statistically significant differences among different cell treatments vs. controls. Also, the co-treatments of different drug concentrations plus H_2_O_2_ were slightly more cytotoxic than the drug alone, with the highest drug concentration plus H_2_O_2_ inducing the lowest cell viability (~72%), yet in a non-statistically significant manner when compared with H_2_O_2_ and the drug alone. The CI value for this combination confirms the presence of slight synergism (Table 5), whereas all other dose–time combinations yielded CI > 1, consistent with an antagonistic interaction. At 48 and 72 h, the cell viability of the combinations and the drug alone was slightly reduced, indicating the presence of antagonism, confirmed by the CI values (CI > 1) (Table 5).

Overall, the presence of an oxidative stress inducer enhanced the efficacy of repurposed drugs, particularly in SH-SY5Y cells. Notably, clozapine’s anticancer activity was potentiated in both cell lines, highlighting its potential as a candidate for glioblastoma and neuroblastoma treatment. These findings suggest that oxidative stress can modulate drug efficacy—either enhancing or reducing it—depending on the cell type, drug concentration, and exposure time.

#### 3.2.3. The Presence of DA Precursor and Oxidative Stress Induces Synergism on SH-SY5Y and A172 Cell Lines

**Figure 7:**
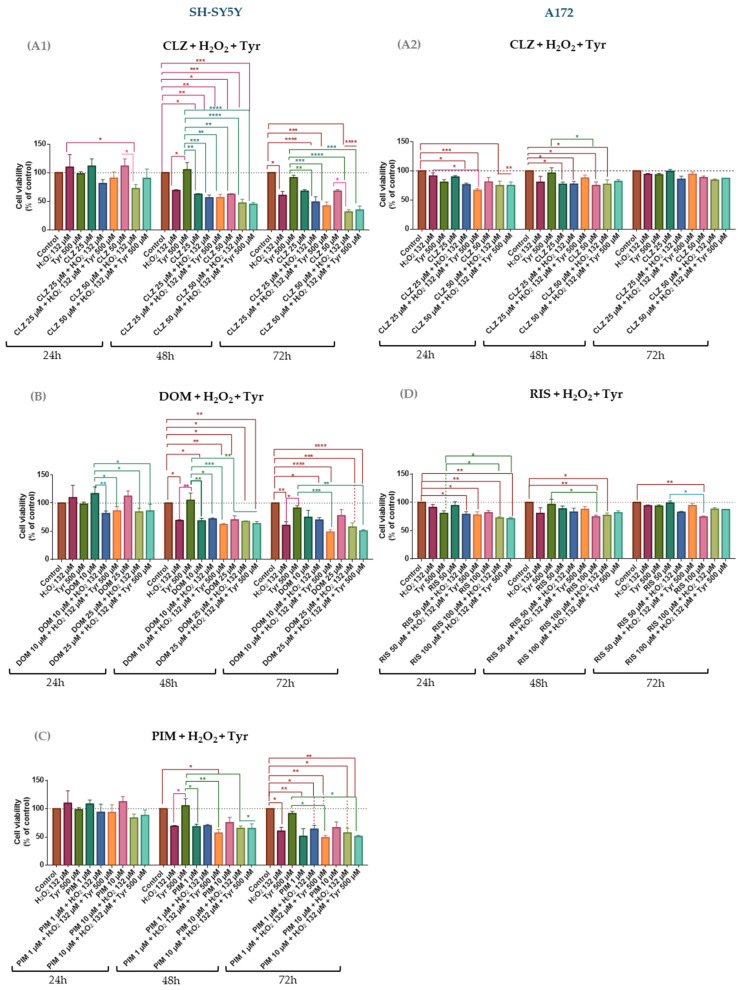
Effect of addition of Tyr, a DA precursor, on CLZ (**A1**,**A2**), DOM (**B**), PIM (**C**), and RIS (**D**) activity with a stress oxidative inducer on the viability of SH-SY5Y and A172 cells, respectively. The cells were cultured in two concentrations of repurposed drugs and with a single concentration of H_2_O_2_ and Tyr and treated for 24, 48, and 72 h. Then, cell viability was determined using the MTT assay. Results are expressed as a percentage of the vehicle-treated control ± SEM of three separate experiments. Statistically significant * *p* < 0.05, ** *p* < 0.01, *** *p* < 0.001, and **** *p* < 0.0001 vs. indicated group.

The effect of the presence of the oxidative stress inducer (H_2_O_2_) and a precursor of DA (Tyr) on drug cytotoxicity was evaluated on SH-SY5Y and A172. Thus, the repurposed drugs were combined with a fixed concentration of H_2_O_2_ and Tyr and incubated for 24, 48, and 72 h. The cell viability for different combinations is depicted in Figure 7, and morphological analyses of cells treated with these combinations are shown in Figures S14–S18.

In SH-SY5Y cells, the combination of clozapine (CLZ) with H_2_O_2_ and tyrosine (Tyr) consistently resulted in lower cell viability compared to CLZ alone, regardless of the drug concentration (Figure 7A1). This reduction was statistically significant for the CLZ 50 µM + H_2_O_2_ combinations and was time-dependent, with the lowest viability observed at 72 h (~31.5%). Interestingly, the addition of Tyr to the CLZ + H_2_O_2_ co-treatment slightly increased cell viability, although not significantly, suggesting a potential neuroprotective effect. For example, at 24 h, cell viability with CLZ 50 µM + H_2_O_2_ was 72%, while the triple combination increased it to 90%. Combination index (CI) values indicated synergism at 72 h for the triple treatment (CI ~ 0.7), whereas at earlier time points, the interaction was strongly antagonistic (CI >> 1) (Table 6).

The combination of DOM with H_2_O_2_ and Tyr was slightly more toxic to SH-SY5Y cells than the combination of DOM with H_2_O_2_, with a statistically significant difference at 24 h for the lowest drug concentration. Increasing time exposure to 48 h leads to a cell viability decrease in all treatments, with the exception of Tyr 500 µM; yet, the differences of cell viability among the drug versus combinations were attenuated, where the cell viability of combinations is similar to the drug alone, and at 72 h, this difference becomes more pronounced, yet in a non-statistically significant manner. For example, at 72 h, the cell viability of DOM 25 µM was ~77.8% in comparison with the drug plus H_2_O_2_ ~57.4% and triple combination ~50.9%. This might indicate that Tyr enhanced toxicity in this combination (Figure 7B), contrary to CLZ (Figure 7A1). The CI values demonstrated synergism in DOM plus H_2_O_2_ and Tyr at 24 h (CI > 1) and an additive effect of co-treatment with DOM 10 µM, H_2_O_2_, and Tyr (Table 6).

Similar results were observed for the combination of PIM with H_2_O_2_ and Tyr. The addition of Tyr increased toxicity, resulting in a decrease in cell viability compared to the combination of PIM with H_2_O_2_ alone, more pronounced at 72 h and the highest drug concentration (Figure 7C). At this time of exposure, the cell viability of PIM 10 µM was ~64.6% in comparison with the drug plus H_2_O_2_ ~57.2% and the triple combination ~50,6%. As previously observed, increased time of exposure to the drugs and combinations led to a decrease in cell viability, suggesting that cytotoxicity is time-dependent. In these combinations, the CI value lower than 1 was obtained at 24 h for the lower drug combination and at 72 h for both conditions evaluated (Table 6), thus demonstrating that H_2_O_2_ and Tyr might potentiate drug cytotoxicity.

The combination of CLZ with H_2_O_2_ and Tyr (Figure 7A2) showed low toxicity in the A172 cell line in comparison with SH-SY5Y. Nevertheless, at 24 h, the cell viability of the co-treatment of CLZ 25 µM, H_2_O_2_, and Tyr was lower (66.6%) than CLZ 25 µM + H_2_O_2_ (76.2%) and CLZ 25 µM (89.4%). This suggests that Tyr might potentiate the cytotoxicity of the drug. Interestingly, increasing the drug concentration to 50 µM did not decrease the cell viability of the combinations in comparison with the drug alone. Also, unlike what was observed in SH-SY5Y, increased time exposure to compounds leads to an increase in cell viability. For example, the cell viability of CLZ 25 µM + H_2_O_2_ + Tyr at 24 h was 66.6% and at 72 h was 94.3%. Increasing the drug concentration led to a similar result. The CI value demonstrated that at 24 h, the combinations presented synergism, while at 48 and 72 h, they were highly antagonistic (Table 7).

The results obtained for the combination of RIS with H_2_O_2_ and Tyr were similar to those observed with CLZ. In general, the combinations presented moderated cytotoxicity, being more significant at 24 h, where synergism was observed for both combinations (Table 7). At this time of exposure, the cellular viability of combinations using different drug concentrations was lower than the drug alone (Figure 7D). This difference was more pronounced using the lowest drug concentration, where the cell viability was 94.5% in comparison with 78.8% of RIS 50 µM plus H_2_O_2_ and 77.61% of the triple combination, yet not statistically significant. Herein, the role of Tyr (neuroprotective vs. cytotoxic) is less evident. As observed with CLZ, the increase in drug concentration did not statistically significantly decrease cell viability, i.e., the cell viability at 72 h of RIS 50 µM + H_2_O_2_ + Tyr was 94.3% versus RIS 100 µM + H_2_O_2_ + Tyr 87.4%. In general, increasing time exposure results in the similar or increased cell viability of triple combinations in comparison with the drug alone, suggesting that in this cell line, Tyr could become neuroprotective over time. Comparing the results described above for 24 h with those obtained at 72 h, the cell viability of the drug alone increased up to 98.9% and 94.3% for the triple combination.

**Figure 8:**
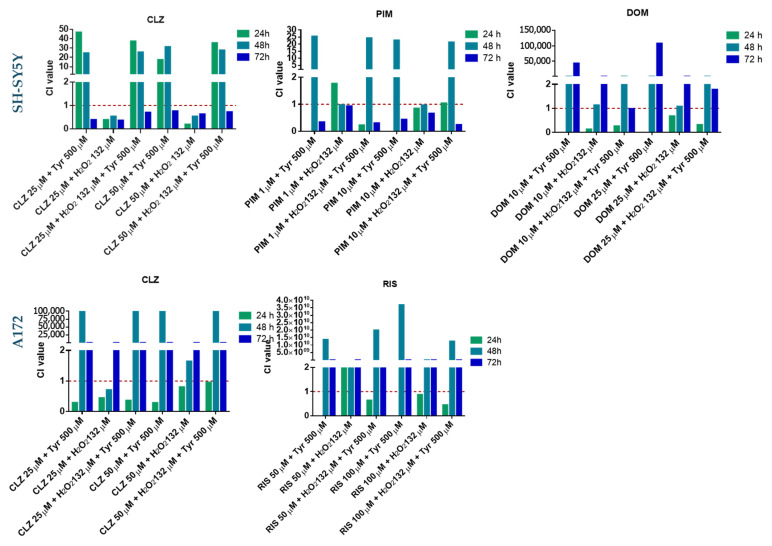
Graphical representation of Combination Index (CI) of repurposed drugs combined with Tyr or H_2_O_2_ alone and the triple combination (Drug + Tyr + H_2_O_2_). The red line (CI =1) defines additivity, lower is synergism (CI < 1), or above is antagonism (CI > 1).

Comparing all the CI values obtained for the combination of different repurposed drugs with H_2_O_2_ (132 µM) and the triple combination (drug + H_2_O_2_ +Tyr 500 µM) demonstrated different tendencies in the two cell lines (Figure 8).

**Figure 9:**
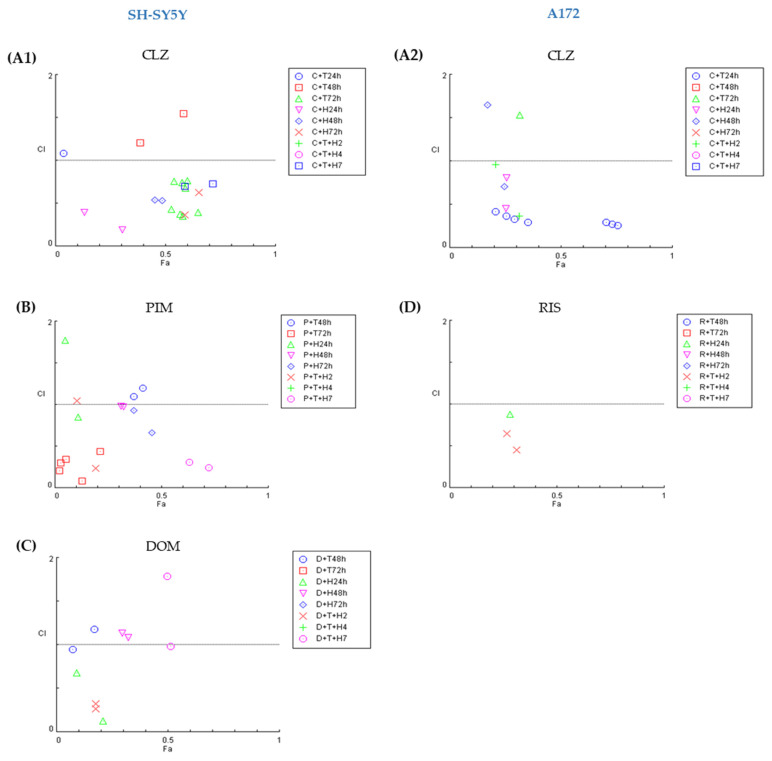
Fa–CI plots of combinations with repurposed drugs (**A1**) CLZ in SH-SY5Y; (**A2**) CLZ in A172; (**B**) PIM in SH-SY5Y; (**C**) DOM in SH-SY5Y and (**D**) RIS in A172 cell line. CLZ combinations are represented as follows: CLZ + Tyr 24 h (blue circle), CLZ + Tyr 48 h (red square), CLZ + Tyr 72 h (green triangle), CLZ + H_2_O_2_ 24 h (pink triangle), CLZ + H_2_O_2_ 48 h (blue diamond), CLZ + H_2_O_2_ 72 h (red cross), CLZ + H_2_O_2_ + Tyr 24 h (green plus sign), CLZ + H_2_O_2_ + Tyr 48 h (pink circle), and CLZ + H_2_O_2_ + Tyr 72 h (blue square). The other repurposed drug combinations are represented as follows: Drug + Tyr 24 h (blue circle), Drug + Tyr 48 h (red square), Drug + Tyr 72 h (green triangle), Drug + H_2_O_2_ 48 h (pink triangle), Drug + H_2_O_2_ 72 h (blue diamond), Drug + H_2_O_2_ + Tyr 24 h (red cross), Drug + H_2_O_2_ + Tyr 48 h (green plus sign), and Drug + H_2_O_2_ + Tyr 72 h (pink circle).

In A172, it is evident that synergism occurs mostly at 24 h, turning into highly antagonistic with the increase in time exposure, and this is valid for the two different repurposed drugs and all conditions tested, except RIS 50 µM plus H_2_O_2_ (Figure 8). For the SH-SY5Y cell line, the CI value tendency varies depending on the repurposed drug. For DOM, the synergism occurs mostly at 24 h for all conditions, while, as observed in the A172 cell line, the increase in time exposure led to antagonism. By contrast, the combinations of CLZ and PIM induce synergism at 72 h. These results suggested that the time of exposure to compounds might influence the CI value. In terms of the number of combinations that present synergism, the Fa-CI plots of all combinations demonstrated that CLZ presented a higher number of combinations with a CI value lower than 1 in both lines (16 in SH-SYHY vs. 12 in A172), followed by combinations with PIM (11) and RIS (6) in SH-SY5Y and A172, respectively (Figure 9).

In summary, CLZ activity is potentiated in the presence of oxidative stress and the precursor of dopamine.

### 3.3. Impacts of Clozapine and Its Combinations with Tyrosine and H_2_O_2_ on Extracellular Tyrosine and Dopamine Levels in SH-SY5Y and A172 Cell Lines

Given that CLZ emerged as the most promising drug in the combinations tested on both cell lines, we then further investigated its effects alone or combined, using uHPLC to observe the potential alterations of concentrations of Tyr and DA. To assess the influence of the 48 h treatment with CLZ and its combinations with Tyr and H_2_O_2_ on extracellular Tyr and DA concentrations in the SH-SY5Y and A172 cell lines, cells were treated with CLZ at concentrations of 25 and 50 µM, and Tyr and H_2_O_2_ at concentrations of 500 and 132 µM, respectively. The high concentration of Tyr was chosen to ensure a measurable response, allowing a more precise interpretation of the HPLC results. DA was not detected in all extracellular mediums analyzed. The results of Tyr detection in extracellular medium by uHPLC analysis in different cell lines are depicted in Table 8 and Table 9. Regarding 48 h of Tyr exposure in SH-SY5Y cells, the concentration of Tyr in the extracellular medium decreased by approximately 147 µM. When Tyr was combined with CLZ at 25 µM, Tyr levels in the extracellular medium decreased by about 115 µM, and with CLZ at 50 µM, Tyr levels decreased by around 207 µM (Table 8). Additionally, no Tyr was detected in any of the samples when Tyr was not added to the medium. Furthermore, adding H_2_O_2_ to the combination of CLZ and Tyr resulted in undetectable Tyr levels, indicating that Tyr was rapidly and completely consumed by the cells, which suggests that Tyr might be important for cellular maintenance.

In A172 cells, the concentration of Tyr in the extracellular medium increased by approximately 86 µM. When combined with CLZ at 25 µM, Tyr levels decreased by about 385 µM, and with CLZ at 50 µM, Tyr levels decreased by around 361 µM (Table 9). When Tyr was combined with CLZ at 25 µM and H_2_O_2_, Tyr levels increased by approximately 103 µM, and with CLZ 50 µM and H_2_O_2_, Tyr levels decreased by around 299 µM (Table 9). Additionally, when Tyr was not added to the medium, no Tyr was detected in any samples. Furthermore, unlike the results in the SH-SY5Y cells, the addition of H_2_O_2_ to the combination of CLZ and Tyr did not result in undetectable levels of Tyr, and one combination even showed higher Tyr levels than the standard.

## 4. Discussion

To investigate whether tyrosine (Tyr), a dopamine (DA) precursor, influences the activity of repurposed drugs with anticancer potential, six dopamine receptor-targeting compounds were initially tested on SH-SY5Y and A172 cell lines. The most cytotoxic drugs in SH-SY5Y cells were clozapine (CLZ), domperidone (DOM), and pimozide (PIM), whereas CLZ and risperidone (RIS) were most effective in A172 cells. These compounds act primarily as dopamine receptor antagonists, reducing DA-mediated signaling. Notably, except for DOM, all drugs with significant anticancer activity are antipsychotics, consistent with prior studies suggesting their potential in oncology [26,35].

Antipsychotic CLZ induced significant cell death in SH-SY5Y and A172 at higher concentrations and more pronounced at 72 h. Previous studies have demonstrated that CLZ alters the metabolic activity and regulates the phosphorylation of GSK-3beta through Wnt signal pathways in SH-SY5Y [42]. The anticancer activity of CLZ in A172 was previously evaluated and demonstrated that the repurposed drug was cytotoxic, which is in line with the results obtained in this study [43]. Notably, CLZ was the only drug among the six tested that showed anticancer effects in both cell lines, highlighting its potent cytotoxic properties for the treatment of central nervous system tumors.

Regarding other active repurposed drugs, PIM demonstrated significant cytotoxicity in line with studies, supporting its potential as an anticancer agent. Its antiproliferative effects have been demonstrated in various tumor cell lines, including neuroblastoma cells [25,26]. Nevertheless, this cytotoxicity was not observed in the A172 cell line, suggesting that the mode of action is cell-type specific. Regarding RIS, some studies have shown that it has anticancer activities in neuroblastoma cells [44]. In SH-SY5Y cells, risperidone (RIS) exhibited a dual response: low concentrations enhanced viability at 24 h—suggesting transient neuroprotection—whereas higher doses and longer exposure (72 h) induced moderate cytotoxicity. In A172 glioblastoma cells, RIS decreased viability at all tested concentrations (significantly at higher doses), consistent with reports that it inhibits proliferation, induces apoptosis [45], and can potentiate temozolomide’s antitumor effects [46]. It has also been demonstrated that RIS potentiated the activity of the first-line chemotherapeutic agent for glioblastoma, temozolomide, by suppressing glioma cell growth [47]. In contrast, OLZ showed no significant cytotoxicity in either cell line. This aligns with reports describing its reductions in tumor cell viability effects in SH-SY5Y cells and animal models [48,49]. Nonetheless, other studies have demonstrated antiproliferative activity of OLZ in A172 cells, with an IC_50_ of 27.9 µM [50], a result not replicated here, where the IC_50_ exceeded 100 µM. This discrepancy may stem from methodological differences, including drug concentration, incubation times, or cell culture conditions.

As a preliminary step before testing combinations, the effect of tyrosine (Tyr) alone was evaluated in both cell lines. Tyr did not exhibit cytotoxicity, which aligns with its physiological role and endogenous production. In glioblastoma (GBM), Tyr metabolism is known to influence tumor growth, invasion, and modulation of the tumor microenvironment. Dysregulation of receptor tyrosine kinase (RTK) pathways—commonly altered in GBM—contributes to enhanced cell proliferation and survival [51]. Although slight, non-significant reductions in cell viability were observed at higher concentrations; these may result from oxidative stress caused by tyrosyl radical formation, as previously described [45]. Moreover, SH-SY5Y cells express enzymes involved in Tyr biosynthesis, further supporting their tolerance to extracellular Tyr [52,53].

Combining Tyr with CLZ enhanced cytotoxicity versus CLZ alone, most notably at 48 h in SH-SY5Y cells and at 24 h and 48 h in A172 cells. In A172, synergy was observed at 50 µM CLZ, while at lower CLZ doses, Tyr appeared to attenuate toxicity, suggesting dose-dependent modulation. The combination index (CI) analysis confirmed synergism in both lines, demonstrating that Tyr potentiates CLZ’s anticancer effect. Elucidating the molecular drivers of this synergy will require targeted pathway investigations. Tyr co-treatment did not enhance—and in some cases reduced—the cytotoxicity of DOM, PIM, and RIS in SH-SY5Y and A172 cells. In SH-SY5Y, DOM + Tyr increased viability at 72 h, consistent with antagonism, while PIM combinations yielded only modest effects, perhaps due to lower tested doses. In A172, Tyr failed to potentiate RIS activity and even suggested antagonism. Testing higher DOM or PIM concentrations may clarify these drug–amino acid interactions. Overall, these findings suggest that combining Tyr with DOM, PIM, or RIS does not improve, and may even impair, their anticancer efficacy. There is evidence of the connection between extracellular amino acid availability and an antipsychotic’s anticancer efficacy of PIM: the drug blocks lysosomal lipid release, GBM cells compensate by up-regulating the glutamine transporter ASCT2, and co-inhibition of glutamine uptake or GLS (e.g., pimozide + CB-839) collapses this feed-forward loop and markedly enhances tumor suppression [54]. Our finding that exogenous tyrosine augments clozapine cytotoxicity extends this principle to the dopamine/tyrosine axis, indicating that amino acid availability can be an actionable determinant of atypical antipsychotic activity in GBM. Although indole-3-acetonitrile (IAN) is not an antipsychotic agent, this metabolite becomes significantly more cytotoxic to SH-SY5Y neuroblastoma cells when co-administered with its amino acid precursors tyrosine or tryptophan, underscoring the broader principle that exogenous amino acids can modulate the activity of neuroactive compounds [55]. Together, these studies reinforce our proposal that manipulating the amino acid supply could be a general strategy to unlock additional antitumor potential in repurposed neuropsychiatric drugs. These insights argue for systematically testing amino acid manipulation alongside newer agents such as brexpiprazole, aripiprazole, and lurasidone in future studies.

Only clozapine (CLZ) showed a slight, non-significant reduction in SH-SY5Y viability under H_2_O_2_-induced stress; yet, combination-index analysis revealed near-zero CI values, indicating strong synergism. These data suggest that oxidative stress amplifies CLZ’s cytotoxicity rather than diminishing it, highlighting its potential utility in malignancies or pathologies marked by high reactive-oxygen species levels. Although extracellular H_2_O_2_ is scavenged rapidly—polarographic assays show >90% of a 100 µM bolus is eliminated within ~60 min by catalase [56]—its downstream damage can persist far longer. Brief exposures of 100–200 µM H_2_O_2_ suppress mesenchymal-stem-cell proliferation for >30 h [57] and trigger progressive neuronal apoptosis up to 24 h post-exposure [58]. Recent studies reported sustained disruption of tryptophan/serotonin metabolism 24 h after an exposure of H_2_O_2_ at 100 µM in SH-SY5Y neuroblastoma cells [59,60]. These observations support our use of 24- and 48-h read-outs to capture the prolonged biological consequences of an initial 132 µM H_2_O_2_ challenge in the drug-combination assays. DOM and PIM showed no significant viability changes under H_2_O_2_: DOM exhibited early (24 h) synergy that shifted to antagonism at later times, likely due to prolonged oxidative damage, while PIM remained nearly additive throughout. In A172 cells, CLZ  +  H_2_O_2_ retained 24 h synergy but RIS combinations were antagonistic, and neither improved viability beyond single agents. Across both lines, CLZ’s enhancement by tyrosine exceeded that by H_2_O_2_, underscoring cell- and context-dependent redox/drug interactions and suggesting GBM’s relative resistance to oxidative priming. This resistance could represent a mechanism by which tumor cells evade therapy under high oxidative stress. The combined presence of Tyr and H_2_O_2_ had distinct, cell-line–specific effects on drug cytotoxicity. In SH-SY5Y cells, adding Tyr to the drug  +  H_2_O_2_ pair did not change viability versus drug  +  H_2_O_2_, but the triple combination reduced viability relative to drug  +  Tyr, with significant CI-defined synergism at 72 h for CLZ and PIM and at 24 h for DOM. This suggests that while Tyr alone mitigates oxidative-stress protection, H_2_O_2_ drives drug potentiation—especially for CLZ. In A172 cells, Tyr addition to drug  +  H_2_O_2_ had minimal impact on viability versus drug  +  H_2_O_2_, displaying early (24 h) synergy that shifted to antagonism over longer exposures, indicative of increasing resistance to sustained oxidative stress. These findings highlight that oxidative and amino-acid cues interact differently across tumor subtypes, emphasizing the need to dissect underlying molecular determinants to optimize combination therapies. Extracellular tyrosine measurements under the CLZ  +  Tyr  +  H_2_O_2_ regimen reveal divergent metabolic adaptations in our two models. In SH-SY5Y cells, triple treatment drove Tyr to undetectable levels—coincident with marked viability loss—suggesting aggressive uptake or catabolism to counter redox stress. By contrast, A172 cells often maintained or even elevated extracellular Tyr (notably at 25 µM CLZ  +  132 µM H_2_O_2_  +  500 µM Tyr), despite reduced viability, indicating a relative inability or strategic choice to export excess amino acid. These patterns imply that neuroblastoma and glioblastoma cells deploy distinct Tyr-centric metabolic responses when faced with combined dopaminergic and oxidative insults, a nuance that may inform tumor-type–specific optimization of repurposed-drug therapies. The consumption of Tyr likely occurred because the cells, under oxidative stress, utilized all the available nutrients and components from the extracellular medium in an attempt to counteract the oxidative stress and restore balance, which is more evident in neuroblastoma cells than in glioblastoma. This aligns with evidence that describes glioblastoma cells as being more resistant to chemotherapeutic and radiation treatment [61]. As SH-SY5Y cells are known to be a valuable tool in research involving DA synthesis [62], no DA concentration was detected in the extracellular medium, contrary to expectations. This absence suggests that DA was either completely consumed by the cells or was present at such low levels that uHPLC could not detect it. These findings further support the idea that SH-SY5Y cells metabolize key compounds rapidly under stress, likely to protect against cellular damage, which contrasts with the more aggressive and resistant A172 cells.

Overall, our results highlight the potential anticancer properties of certain repurposed drugs targeting dopamine receptors, particularly antipsychotics, in neuro-oncology, while also highlighting their behavior in combination with an amino acid and under conditions of cellular stress.

## 5. Conclusions

In this study, we demonstrated that several repurposed dopaminergic agents exert cytotoxic effects on neuroblastoma (SH-SY5Y) and glioblastoma (A172) cell lines, with clozapine (CLZ) showing the strongest single-agent activity and the most consistent synergism in combination with L-tyrosine or oxidative stress (H_2_O_2_) across both models. The differential responses observed for domperidone, pimozide, and risperidone underscore the importance of receptor affinity and cell-type specificity in mediating anticancer effects.

These results provide a strong rationale for further in vivo studies to validate the anticancer potential of CLZ in combination with Tyr, especially in tumors of dopaminergic origin or those with dysregulated amino acid metabolism. The CLZ unique receptor binding profile—characterized by high affinity for dopamine D_4_ receptors (K_i_ < 10 nM), but low affinity for D_2_ [32,33,34]—may underlie its distinctive cytotoxic and synergistic effects observed in this study, particularly under conditions of oxidative stress or presence of tyrosine.

We acknowledge that the concentrations employed here exceed those achievable in clinical settings. Thus, this work should be regarded as a proof-of-concept that tyrosine availability and oxidative status can modulate clozapine’s anticancer activity. Future studies should (i) evaluate lower, clinically relevant dosing regimens; (ii) profile dopamine receptor expression (particularly D_4_ vs. D_2_) in tumor cells to clarify the mechanism of action; and (iii) validate the efficacy and safety in in vivo models before considering translation to neuro-oncology applications and exploring tumor metabolic signatures. Thus, the validation of the most promising findings through in silico analysis followed by in vivo studies would be essential to confirm their clinical applicability.

## Figures and Tables

**Table 1:** Half-maximal inhibitory concentration (IC_50_) values of the six repurposed drugs in SH-SY5Y and A172 cells.

	IC_50_ (µM)					
	SH-SY5			A172		
	24 h	48 h	72 h	24 h	48 h	72 h
Domperidone	30.3	18.6	17.6	>100	>100	>100
Droperidol	>100	>100	>100	>100	>100	>100
Pimozide	6.6	1.8	2.9	>100	>100	>100
Risperidone	>100	>100	>100	>100	51.6	>100
Clozapine	41	31.4	27.4	50.8	42.2	41.3
Olanzapine	>100	>100	>100	>100	>100	>100

**Table 2:** Combination index (CI) values of CLZ and Tyr combinations in SH-SY5Y and A172 cells at 24, 48, and 72 h. CI < 1 indicates synergism, CI = 1 indicates additivity, and CI > 1 indicates antagonism.

		SH-SY5Y			A172		
[CLZ] (µM)	[Tyr] (µM)	CI (24 h)	CI (48 h)	CI (72 h)	CI (24 h)	CI (48 h)	CI (72 h)
25	25	1.085	1.209	0.3500	0.3634	8.23 × 10^8^	84.66
25	100	2.844	4.400	0.3731	0.3330	7.715 × 10^8^	70.80
25	250	15.19	11.52	0.4312	0.4137	7.82 × 10^8^	20.48
25	500	46.87	24.72	0.3963	0.2922	9.89 × 10^9^	8.721
50	25	595.6	1.544	0.6803	0.2576	1.30 × 10^11^	1.535
50	100	595.6	6.141	0.7595	0.2730	3.68 × 10^11^	7.658
50	250	595.6	14.81	0.7454	0.2737	1.73 × 10^12^	3.040
50	500	17.48	31.40	0.7630	0.2912	2.87 × 10^12^	6.109

**Table 3:** Combination index (CI) values of PIM, DOM, and Ris plus Tyr in SH-SY5Y and A172 cells at 24, 48, and 72 h: CI < 1 indicates synergism, CI = 1 indicates additivity, and CI > 1 indicates antagonism.

	Cell Line	[Drug] (µM)	[Tyr] (µM)	CI (48 h)	CI (72 h)
PIM	SH-SY5Y	1	25	1.100	0.2064
		1	100	4.122	0.08279
		1	250	11.15	0.3007
		1	500	25.47	0.3439
		10	25	1.199	31,900.4
		10	100	3.921	31,901.8
		10	250	11.08	31,904.7
		10	500	22.71	0.4406
DOM		10	25	0.9455	42,935.0
		10	100	2.910	42,936.5
		10	250	8.217	42,939.4
		10	500	19.93	42,944.3
		25	25	1.174	107,337
		25	100	3.387	107,338
		25	250	7.540	107,341
		25	500	17.1888	107,346
RIS	A172	50	25	7.145 × 10^8^	52.01
		50	100	5.613 × 10^8^	422.1
		50	250	6.455 × 10^9^	33.39
		50	500	1.37 × 10^10^	671.6
		100	25	2.485 × 10^8^	5.299 × 10^7^
		100	100	6.387 × 10^8^	3.94 × 10^52^
		100	250	1.33 × 10^10^	9.244 × 10^6^
		100	500	3.70 × 10^10^	2.931 × 10^7^

**Table 4:** Combination index (CI) values of CLZ, PIM, DOM, and H_2_O_2_ combinations in SH-SY5Y and A172 cells at 24, 48, and 72 h: CI < 1 indicates synergism, CI = 1 indicates additivity, and CI > 1 indicates antagonism.

			SH-SY5Y		
	[Drug] (µM)	[H_2_O_2_] (µM)	CI (24 h)	CI (48 h)	CI (72 h)
CLZ	25	132	0.3961	0.5372	0.3657
	50		0.1936	0.5357	0.6272
DOM	10		0.1301	1.135	2.048
	25		0.6782	1.080	2.882
PIM	1		1.770	0.9803	0.9344
	10		0.8500	0.9743	0.6664

**Table 5:** Combination index (CI) values of CLZ, RIS, and H_2_O_2_ combinations in A172 cells at 24, 48, and 72 h: CI < 1 indicates synergism, CI = 1 indicates additivity, and CI > 1 indicates antagonism.

			A172		
	[Drug] (µM)	[H_2_O_2_] (µM)	CI (24 h)	CI (48 h)	CI (72 h)
CLZ	25	132	0.4525	0.7099	8.695
	50		0.8073	1.649	15.53
RIS	50		2.301	3.716	14.45
	100		0.8770	267.5	1.052E5

**Table 6:** Combination index (CI) values of CLZ, PIM, and DOM plus H_2_O_2_ and Tyr in SH-SY5Y cells at 24, 48, and 72 h: CI < 1 indicates synergism, CI = 1 indicates additivity, and CI > 1 indicates antagonism.

	[Drug] (µM)	[H_2_O_2_] (µM)	[Tyr] (µM)	SH-SY5Y		
				CI (24 h)	CI (48 h)	CI (72 h)
CLZ	25	132	500	37.3339	25.6252	0.70109
	50			35.4844	27.7150	0.72783
DOM	10			0.26510	22.9817	0.98122
	25			0.32063	22.2473	1.78671
PIM	1			0.23416	24.2442	0.30503
	10			1.04354	21.3904	0.24447

**Table 7:** Combination index (CI) values of CLZ and RIS, H_2_O_2_, and Tyr combinations in A172 cells at 24, 48, and 72 h: CI < 1 indicates synergism, CI = 1 indicates additivity, and CI > 1 indicates antagonism.

	[Drug] (µM)	[H_2_O_2_] (µM)	[Tyr] (µM)	A172		
				CI (24 h)	CI (48 h)	CI (72 h)
CLZ	25	132	500	0.36817	1.09 × 10^10^	190.077
	50			0.95749	1.41 × 10^10^	33.3389
RIS	50			0.64749	2.00 × 10^10^	9.65919
	100			0.45423	1.25 × 10^10^	16.7132

**Table 8:** Retention times and areas under the curve used to calculate the variation in Tyr concentrations in the cell medium of CLZ and its combinations in SH-SY5Y cells, determined by uHPLC.

SH-SY5Y Cells (48 h)	Retention Time (min)	Total Area	Tyr Area	Final Area	[Tyr] (µM)
Standard	2.694	-	195,139.56	-	-
Control	2.884	-	-	-	-
Tyr 500 µM	2.905	234,969.88	91,406.03	103,733.53	353.19
CLZ 25 µM	2.728	-	-	-	-
CLZ 25 µM + Tyr 500 µM	2.690	225,583.15	82,019.33	113,120.23	385.22
CLZ 25 µM + H_2_O_2_ 132 µM	2.679	-	-	-	-
CLZ 25 µM + H_2_O_2_ 132 µM + Tyr 500 µM	2.679	143,483.79	−80.3	Tyr was all consumed	-
CLZ 50 µM	2.684	-	-	-	-
CLZ 50 µM + Tyr 500 µM	2.677	252,686.25	109,122.43	86,017.13	292.73
CLZ 50 µM + H_2_O_2_ 132 µM	2.709	-	-	-	-
CLZ 50 µM + H_2_O_2_ 132 µM + Tyr 500 µM	2.684	137,808.98	−5754.84	Tyr was all consumed	-

**Table 9:** Retention times and areas under the curve are used to calculate variation in Tyr concentrations in the cell medium of CLZ and its combinations in A172 cells, determined by HPLC.

A172 Cells (48 h)	Retention Time (min)	Total Area	Tyr Area	Final Area	[Tyr] (µM)
Standard	2.694	-	195,139.56	-	-
Control	2.721	-	-	-	-
Tyr 500 µM	2.702	332,258.91	220,009.74	−24,870.18	−85.69
CLZ 25 µM	3.021	-	-	-	-
CLZ 25 µM + Tyr 500 µM	2.681	274,430.57	162,181.4	32,958.16	111.65
CLZ 25 µM + H_2_O_2_ 132 µM	2.714	-	-	-	-
CLZ 25 µM + H_2_O_2_ 132 µM + Tyr 500 µM	2.706	294,476.33	182,227.16	176,912.4	602.93
CLZ 50 µM	2.704	-	-	-	-
CLZ 50 µM + Tyr 500 µM	2.687	266,350.71	154,101.54	41,038.02	139.23
CLZ 50 µM + H_2_O_2_ 132 µM	2.692	-	-	-	-
CLZ 50 µM + H_2_O_2_ 132 µM + Tyr 500 µM	2.700	171,386.49	59,137.32	59,137.32	200.99

## Data Availability

The original contributions presented in the study are included in the article/Supplementary Material, further inquiries can be directed to the corresponding author.

## Supplementary Materials

The following supporting information can be downloaded at: https://www.mdpi.com/article/10.3390/life15071097/s1, Figure S1: Microscopic visualization of the effects of CLZ, DOM, DRO, OLZ, PIM and RIS on the morphology of SH-SY5Y cells over 24, 48 and 72 h. Cells were treated with (**A**,**I**,**Q**) 0.1% DMSO (control), (**B**,**J**,**R**) 0.01 μM, (**C**,**K**,**S**) 0.1 μM, (**D**,**L**,**T**) 1 μM, (**E**,**M**,**U**) 10 μM, (**F**,**N**,**V**) 25 μM, (**G**,**O**,**W**) 50 μM and (**H**,**P**,**X**) 100μM of CLZ, DOM, DRO, OLZ, PIM and RIS. Representative images were obtained with a high contrast (10×) bright field objective (LionHeart FX Automated Microscope) from three independent experiments. Scale bar: 50 μm; Figure S2: Microscopic visualization of the effects of CLZ, DOM, DRO, OLZ, PIM and RIS on the morphology of A172 cells over 24, 48 and 72 h. Cells were treated with (**A**,**I**,**Q**) 0.1% DMSO (control), (**B**,**J**,**R**) 0.01 μM, (**C**,**K**,**S**) 0.1 μM, (**D**,**L**,**T**) 1 μM, (**E**,**M**,**U**) 10 μM, (**F**,**N**,**V**) 25 μM, (**G**,**O**,**W**) 50 μM and (**H**,**P**,**X**) 100 μM of CLZ, DOM, DRO, OLZ, PIM and RIS. Representative images were obtained with a high contrast (10×) bright field objective (LionHeart FX Automated Microscope) from three independent experiments. Scale bar: 50 μm; Figure S3: Microscopic visualization of the effects of Tyrosine on the morphology of SHSY5Y and A172 cells over 24, 48 and 72 h. Cells were treatedwith (**A**,**F**,**K**) 1% Water (control), (**B**,**G**,**L**) 25μM, (**C**,**H**,**M**) 100μM, (**D**,**I**,**N**) 250 μM and (**E**,**J**,**O**) 500μM of Tyr. Representative images were obtained with a high contrast (10×) brightfield objective (LionHeartFX AutomatedMicroscope) from three independent experiments. Scale bar: 50μm. Figure S4: Microscopic visualization of the effects of CLZ combined with Tyr on the morphology of SH-SY5Y cells over 24, 48 and 72 h. Cells were treated with (**A**,**J**,**S**) 0.1% DMSO (control), (**B**,**K**,**T**) CLZ 25 μM + Tyr 25 μM, (**C**,**L**,**U**) CLZ 25 μM + Tyr 100 μM, (**D**,**M**,**V**) CLZ 25 μM + Tyr 250 μM, (**E**,**N**,**W**) CLZ 25 μM + Tyr 500 μM, (**F**,**O**,**X**) CLZ 50 μM + Tyr 25 μM, (**G**,**P**,**Y**) CLZ 50 μM + Tyr 100 μM, (**H**,**Q**,**Z**) CLZ 50 μM + Tyr 250 μM and (**I**,**R**,**AA**) CLZ 50 μM + Tyr 500 μM. Representative images were obtained with a high contrast (10×) bright field objective (LionHeart FX Automated Microscope) from three independent experiments. Scale bar: 50 μm; Figure S5: Microscopic visualization of the effects of DOM combined with Tyr on the morphology of SH-SY5Y cells over 48 and 72 h. Cells were treated with (**A**,**J**,**S**) 0.1% DMSO (control), (**B**,**K**,**T**) DOM 10 μM + Tyr 25 μM, (**C**,**L**,**U**) DOM 10 μM + Tyr 100 μM, (**D**,**M**,**V**) DOM 10 μM + Tyr 250 μM, (**E**,**N**,**W**) DOM 10 μM + Tyr 500 μM, (**F**,**O**,**X**) DOM 25 μM + Tyr 25 μM, (**G**,**P**,**Y**) DOM 25 μM + Tyr 100 μM, (**H**,**Q**,**Z**) DOM 25 μM + Tyr 250 μM and (**I**,**R**,**AA**) DOM 25 μM + Tyr 500 μM. Representative images were obtained with a high contrast (10×) bright field objective (LionHeart FX Automated Microscope) from three independent experiments. Scale bar: 50 μm; Figure S6: Microscopic visualization of the effects of PIM combined with Tyr on the morphology of SH-SY5Y cells over 48 and 72 h. Cells were treated with (**A**,**J**,**S**) 0.1% DMSO (control), (**B**,**K**,**T**) PIM 1 μM + Tyr 25 μM, (**C**,**L**,**U**) PIM 1 μM + Tyr 100 μM, (**D**,**M**,**V**) PIM 1 μM + Tyr 250 μM, (**E**,**N**,**W**) PIM 1 μM + Tyr 500 μM, (**F**,**O**,**X**) PIM 10 μM + Tyr 25 μM, (**G**,**P**,**Y**) PIM 10 μM + Tyr 100 μM, (**H**,**Q**,**Z**) PIM 10 μM + Tyr 250 μM and (**I**,**R**,**AA**) PIM 10 μM + Tyr 500 μM. Representative images were obtained with a high contrast (10×) bright field objective (LionHeart FX Automated Microscope) from three independent experiments. Scale bar: 50 μm; Figure S7: Microscopic visualization of the effects of CLZ combined with Tyr on the morphology of A172 cells over 24, 48 and 72 h. Cells were treated with (**A**,**J**,**S**) 0.1% DMSO (control), (**B**,**K**,**T**) CLZ 25 μM + Tyr 25 μM, (**C**,**L**,**U**) CLZ 25 μM + Tyr 100 μM, (**D**,**M**,**V**) CLZ 25 μM + Tyr 250 μM, (**E**,**N**,**W**) CLZ 25 μM + Tyr 500 μM, (**F**,**O**,**X**) CLZ 50 μM + Tyr 25 μM, (**G**,**P**,**Y**) CLZ 50 μM + Tyr 100 μM, (**H**,**Q**,**Z**) CLZ 50 μM + Tyr 250 μM and (**I**,**R**,**AA**) CLZ 50 μM + Tyr 500 μM. Representative images were obtained with a high contrast (10×) bright field objective (LionHeart FX Automated Microscope) from three independent experiments. Scale bar: 50 μm. Figure S8: Microscopic visualization of the effects of RIS combined with Tyr on the morphology of A172 cells over 48 and 72 h. Cells were treated with (**A**,**J**,**S**) 0.1% DMSO (control), (**B**,**K**,**T**) RIS 50 μM + Tyr 25 μM, (**C**,**L**,**U**) RIS 50 μM + Tyr 100 μM, (**D**,**M**,**V**) RIS 50 μM + Tyr 250 μM, (**E**,**N**,**W**) RIS 50 μM + Tyr 500 μM, (**F**,**O**,**X)** RIS 100 μM + Tyr 25 μM, (**G**,**P**,**Y**) RIS 100 μM + Tyr 100 μM, (**H**,**Q**,**Z**) RIS 100 μM + Tyr 250 μM and (**I**,**R**,**AA**) RIS 100 μM + Tyr 500 μM. Representative images were obtained with a high contrast (10×) bright field objective (LionHeart FX Automated Microscope) from three independent experiments. Scale bar: 50 μm; Figure S9: Microscopic visualization of the effects of CLZ combined with H_2_O_2_ on the morphology of SH-SY5Y cells over 24, 48 and 72 h. Cells were treated with (**A**,**E**,**I**) 0.1% DMSO (control), (**B**,**F**,**J**) H2O2 132 μM, (**C**,**L**,**U**) CLZ 25 μM + H_2_O_2_ 132 μM and (**D**,**M**,**V**) CLZ 50 μM + H_2_O_2_ 132 μM. Representative images were obtained with a high contrast (10×) bright field objective (LionHeart FX Automated Microscope) from three independent experiments. Scale bar: 50 μm; Figure S10: Microscopic visualization of the effects of DOM combined with H_2_O_2_ on the morphology of SH-SY5Y cells over 24, 48 and 72 h. Cells were treated with (**A**,**E**,**I**) 0.1% DMSO (control), (**B**,**F**,**J**) H_2_O_2_ 132 μM, (**C**,**L**,**U**) DOM 10 μM + H_2_O_2_ 132 μM and (**D**,**M**,**V**) DOM 25 μM + H_2_O_2_ 132 μM. Representative images were obtained with a high contrast (10×) bright field objective (LionHeart FX Automated Microscope) from three independent experiments. Scale bar: 50 μm; Figure S11: Microscopic visualization of the effects of PIM combined with H_2_O_2_ on the morphology of SH-SY5Y cells over 24, 48 and 72 h. Cells were treated with (**A**,**E**,**I**) 0.1% DMSO (control), (**B**,**F**,**J**) H2O2 132 μM, (**C**,**L**,**U**) PIM 1 μM + H2O2 132 μM and (**D**,**M**,**V**) PIM 10 μM + H_2_O_2_ 132 μM. Representative images were obtained with a high contrast (10×) bright field objective (LionHeart FX Automated Microscope) from three independent experiments. Scale bar: 50 μm; Figure S12. Microscopic visualization of the effects of CLZ combined with H2O2 on the morphology of A172 cells over 24, 48 and 72 h. Cells were treated with (**A**,**E**,**I**) 0.1% DMSO (control), (**B**,**F**,**J**) H_2_O_2_ 132 μM, (**C**,**L**,**U**) CLZ 25 μM + H_2_O_2_ 132 μM and (**D**,**M**,**V**) CLZ 50 μM + H_2_O_2_ 132 μM. Representative images were obtained with a high contrast (10×) bright field objective (LionHeart FX Automated Microscope) from three independent experiments. Scale bar: 50 μm; Figure S13: Microscopic visualization of the effects of RIS combined with H_2_O_2_ on the morphology of A172 cells over 24, 48 and 72 h. Cells were treated with (**A**,**E**,**I**) 0.1% DMSO (control), (**B**,**F**,**J**) H2O2 132 μM, (**C**,**G**,**K**) RIS 50 μM+ H_2_O_2_ 132 μM and (**D**,**H**,**L**) RIS 100 μM + H2O2 132 μM. Representative images were obtained with a high contrast (10×) bright field objective (LionHeart FX Automated Microscope) from three independent experiments. Scale bar: 50 μm; Figure S14: Microscopic visualization of the effects of CLZ combined with H_2_O_2_ and with Tyr on the morphology of A172 cells over 24, 48 and 72 h. Cells were treated with (**A**,**D**,**G**) 0.1% DMSO (control), (**B**,**E**,**H**) CLZ 25 μM + H_2_O_2_ 132 μM + Tyr 500 μM and (**C**,**F**,**I**) CLZ 50 μM + H_2_O_2_ 132 μM + Tyr 500 μM. Representative images were obtained with a high contrast (10×) bright field objective (LionHeart FX Automated Microscope) from three independent experiments. Scale bar: 50 μm; Figure S15: Microscopic visualization of the effects of DOM combined with H_2_O_2_ and with Tyr on the morphology of A172 cells over 24, 48 and 72 h. Cells were treated with (**A**,**D**,**G**) 0.1% DMSO (control), (**B**,**E**,**H**) DOM 10 μM + H_2_O_2_ 132 μM + Tyr 500 μM and (**C**,**F**,**I**) DOM 25 μM + H_2_O_2_ 132 μM + Tyr 500 μM. Representative images were obtained with a high contrast (10×) bright field objective (LionHeart FX Automated Microscope) from three independent experiments. Scale bar: 50 μm; Figure S16: Microscopic visualization of the effects of PIM combined with H2O2 and with Tyr on the morphology of A172 cells over 24, 48 and 72 h. Cells were treated with (**A**,**D**,**G**) 0.1% DMSO (control), (**B**,**E**,**H**) PIM 1 μM + H_2_O_2_ 132 μM + Tyr 500 μM and (**C**,**F**,**I**) PIM 10 μM + H_2_O_2_ 132 μM + Tyr 500 μM. Representative images were obtained with a high contrast (10×) bright field objective (LionHeart FX Automated Microscope) from three independent experiments. Scale bar: 50 μm; Figure S17: Microscopic visualization of the effects of CLZ combined with H_2_O_2_ and with Tyr on the morphology of A172 cells over 24, 48 and 72 h. Cells were treated with (**A**,**D**,**G**) 0.1% DMSO (control), (**B**,**E**,**H**) CLZ 25 μM + H_2_O_2_ 132 μM + Tyr 500 μM and (**C**,**F**,**I**) CLZ 50 μM + H_2_O_2_ 132 μM + Tyr 500 μM. Representative images were obtained with a high contrast (10×) bright field objective (LionHeart FX Automated Microscope) from three independent experiments. Scale bar: 50 μm; Figure S18: Microscopic visualization of the effects of RIS combined with H_2_O_2_ and with Tyr on the morphology of A172 cells over 24, 48 and 72 h. Cells were treated with (**A**,**E**,**I**) 0.1% DMSO (control), (**B**,**E**,**H**) RIS 50 μM + H_2_O_2_ 132 μM + Tyr 500 μM and (**C**,**F**,**I**) RIS 100 μM + H_2_O_2_ 132 μM + Tyr 500 μM. Representative images were obtained with a high contrast (10×) bright field objective (LionHeart FX Automated Microscope) from three independent experiments. Scale bar: 50 μm.
