# Supplementary material for: Repurposed Antipsychotics as Potential Anticancer Agents: Clozapine Efficacy and Dopaminergic Pathways in Neuroblastoma and Glioblastoma
Source: Life (Basel). 2025 Jul 12;15(7):1097. doi: 10.3390/life15071097 (PMC12298582; doi:10.3390/life15071097)
Supplement: Supplementary file 1 [file life-15-01097-s001.zip › life-3720235-supplementary.pdf]

Supplementary Materials

Repurposed Antipsychotics as Potential Anticancer Agents: Clozapine Efficacy and Dopaminergic Pathways in Neuroblastoma and Glioblastoma

Catarina Moura [1,2], Maria João Gouveia [1] and Nuno Vale [1,3,4,*]

1 PerMed Research Group, RISE-Health, Faculty of Medicine, University of Porto, Alameda Professor Hernâni Monteiro, 4200-319 Porto, Portugal; cafsm13@gmail.com (C.M.); mariajoaogouveia@gmail.com (M.J.G.)

2 ICBAS—School of Medicine and Biomedical Sciences, University of Porto, Rua Jorge Viterbo Ferreira, 228, 4050-313 Porto, Portugal

3 RISE-Health, Department of Community Medicine, Health Information and Decision (MEDCIDS), Faculty of Medicine, University of Porto, Rua Doutor Plácido da Costa, 4200-450 Porto, Portugal

4 Department of Community Medicine, Information and Health Decision Sciences (MEDCIDS), Faculty of Medicine, University of Porto, Rua Doutor Plácido da Costa, 4200-450 Porto, Portugal

* Correspondence: nunovale@med.up.pt; Tel.: +351-220426537

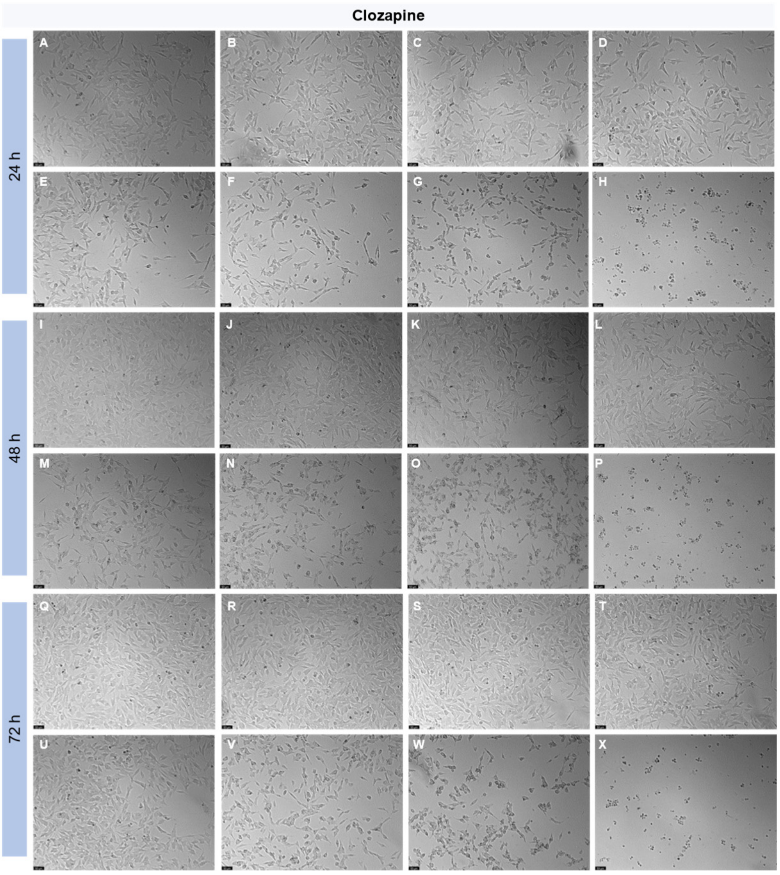

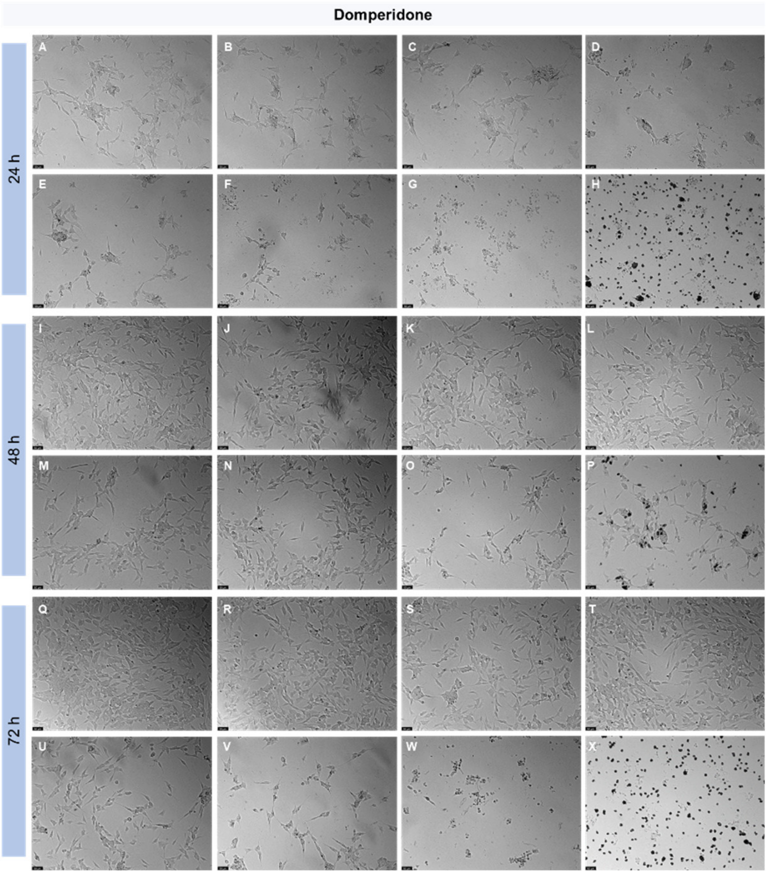
Domperidone
24 h
48 h
72 h

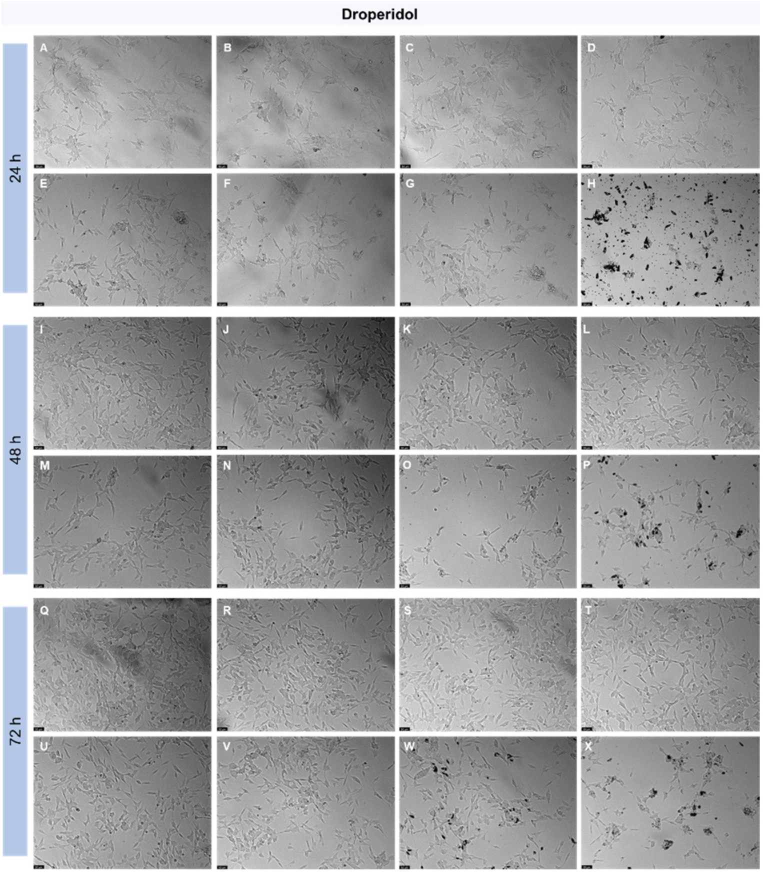
Droperidol
24 h
48 h
72 h

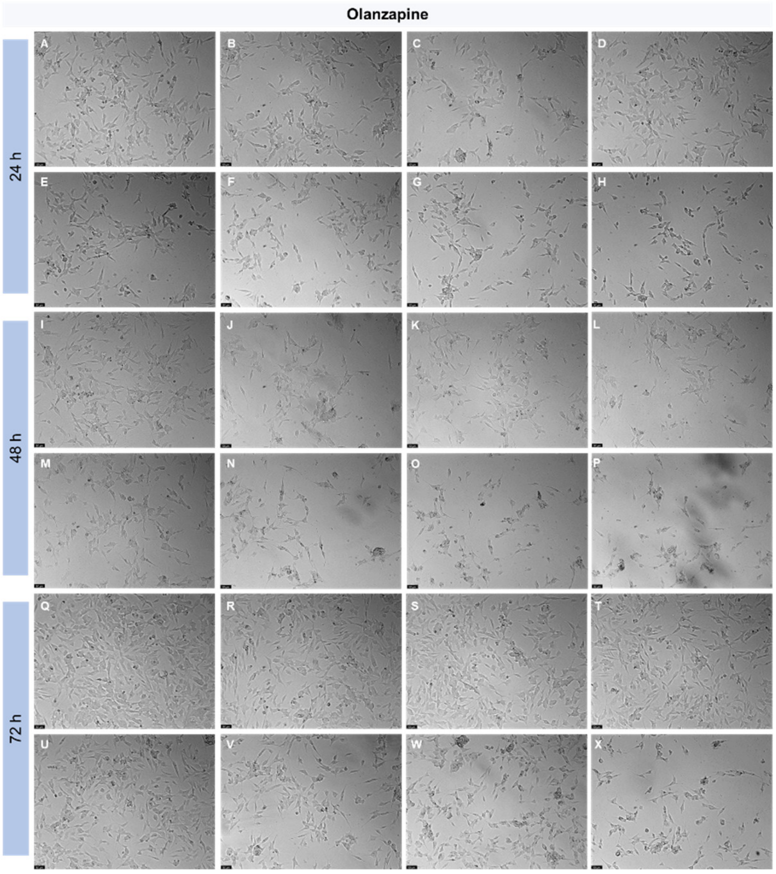
Olanzapine
24 h
48 h
72 h
A
B
C
D
E
F
G
H
I
J
K
L
M
N
O
P
Q
R
S
T
U
V
W
X

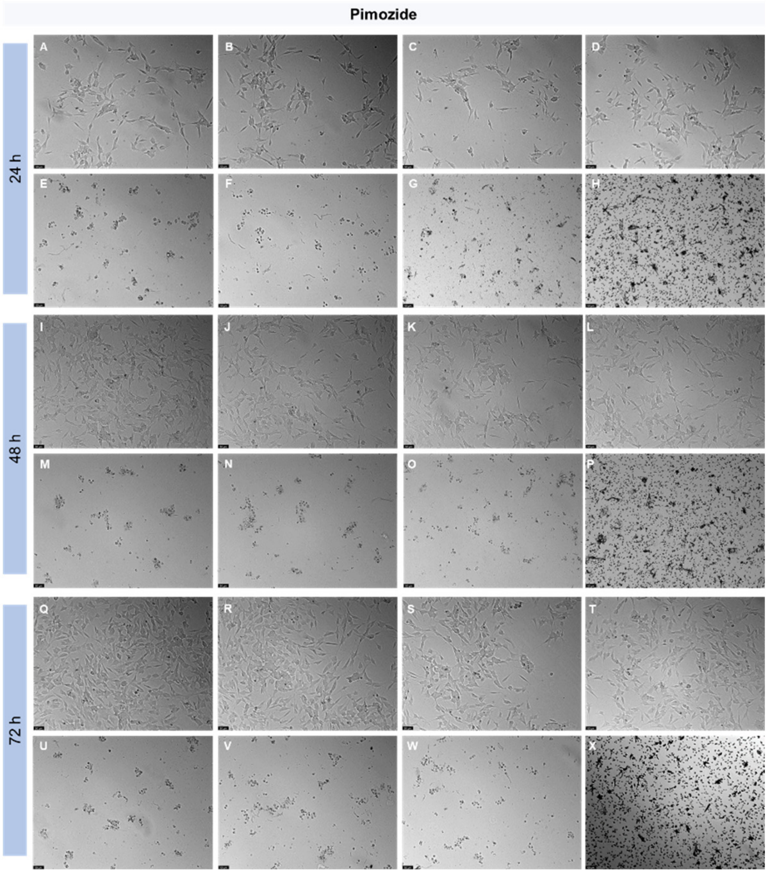
Pimozide
24 h
48 h
72 h
A
B
C
D
E
F
G
H
I
J
K
L
M
N
O
P
Q
R
S
T
U
V
W
X

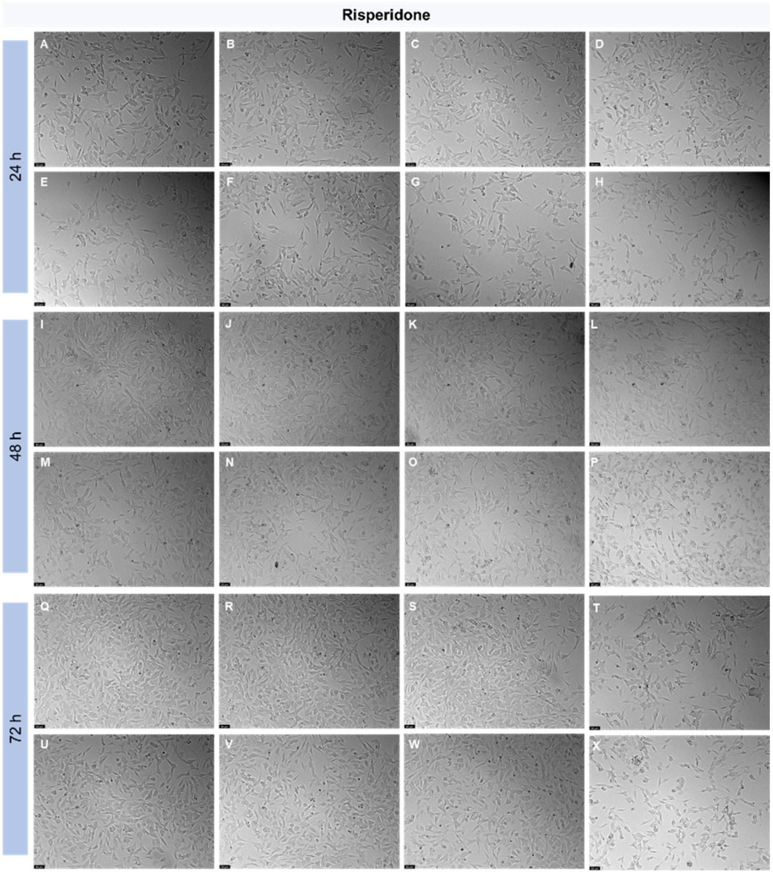

Figure S1. Microscopic visualization of the effects of CLZ, DOM, DRO, OLZ, PIM and RIS on the morphology of SH-SY5Y cells over 24, 48 and 72 hours. Cells were treated with (**A,I,Q**) 0.1% DMSO (control), (**B,J,R**) 0.01 µM, (**C,K,S**) 0.1 µM, (**D,L,T**) 1 µM, (**E,M,U**) 10 µM, (**F,N,V**) 25 µM, (**G,O,W**) 50 µM and (**H,P,X**) 100µM of CLZ, DOM, DRO, OLZ, PIM and RIS. Representative images were obtained with a high contrast (10×) bright field objective (LionHeart FX Automated Microscope) from three independent experiments. Scale bar: 50 µm.

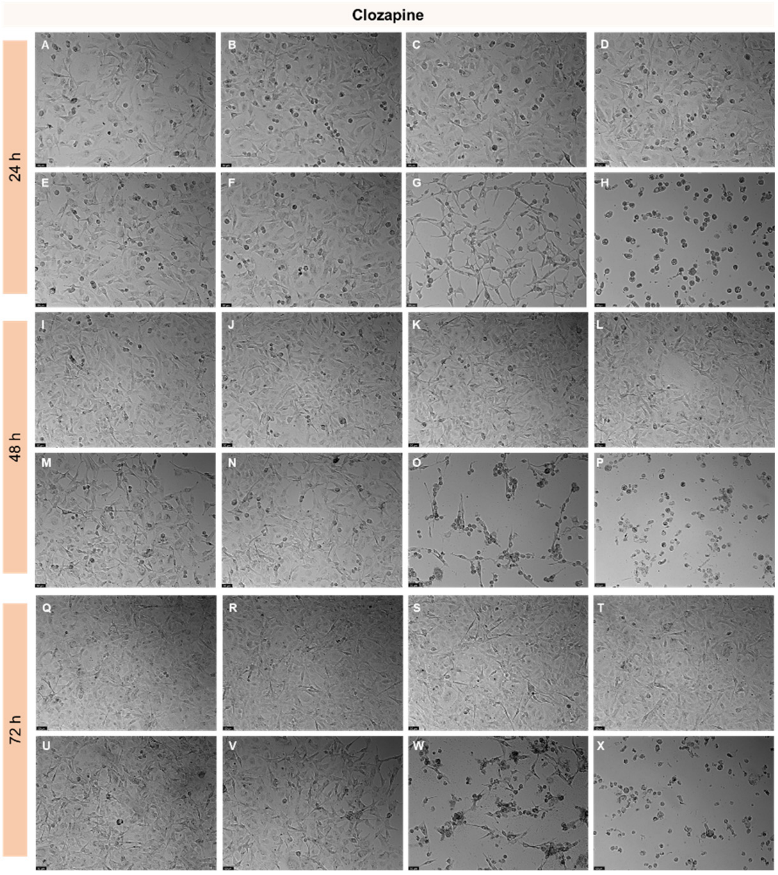
Clozapine
24 h
48 h
72 h
A
B
C
D
E
F
G
H
I
J
K
L
M
N
O
P
Q
R
S
T
U
V
W
X

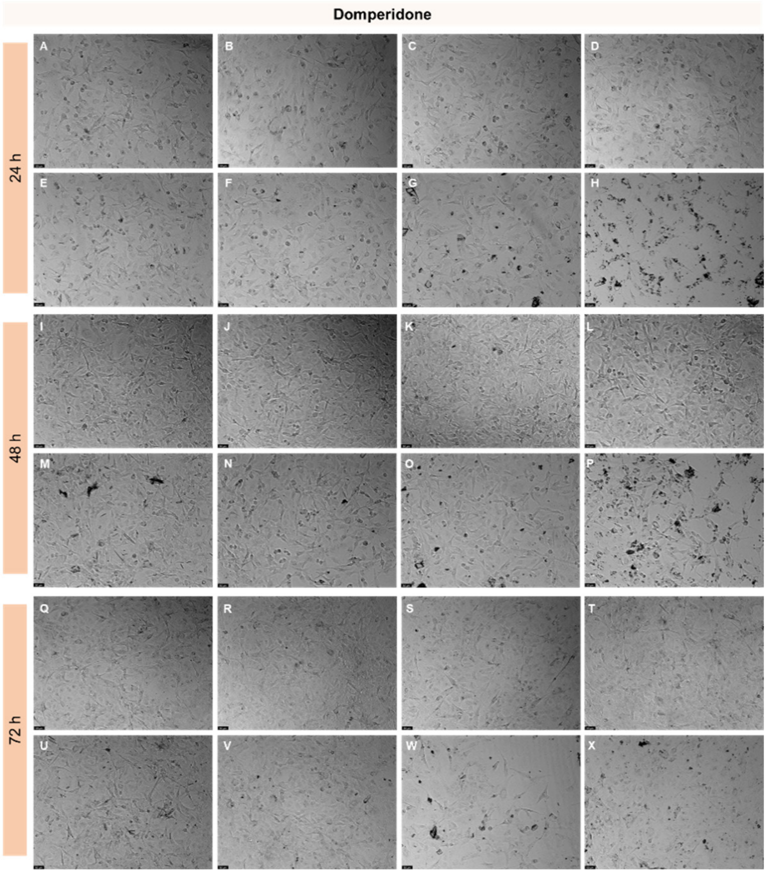
Domperidone
24 h
48 h
72 h
A
B
C
D
E
F
G
H
I
J
K
L
M
N
O
P
Q
R
S
T
U
V
W
X

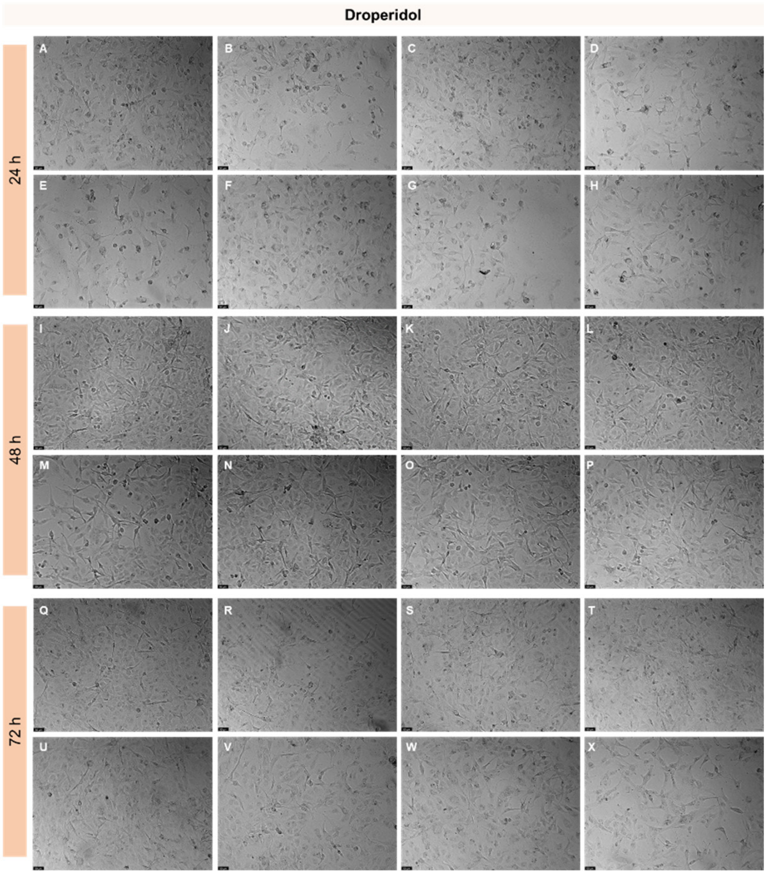
Droperidol
24 h
48 h
72 h
A
B
C
D
E
F
G
H
I
J
K
L
M
N
O
P
Q
R
S
T
U
V
W
X

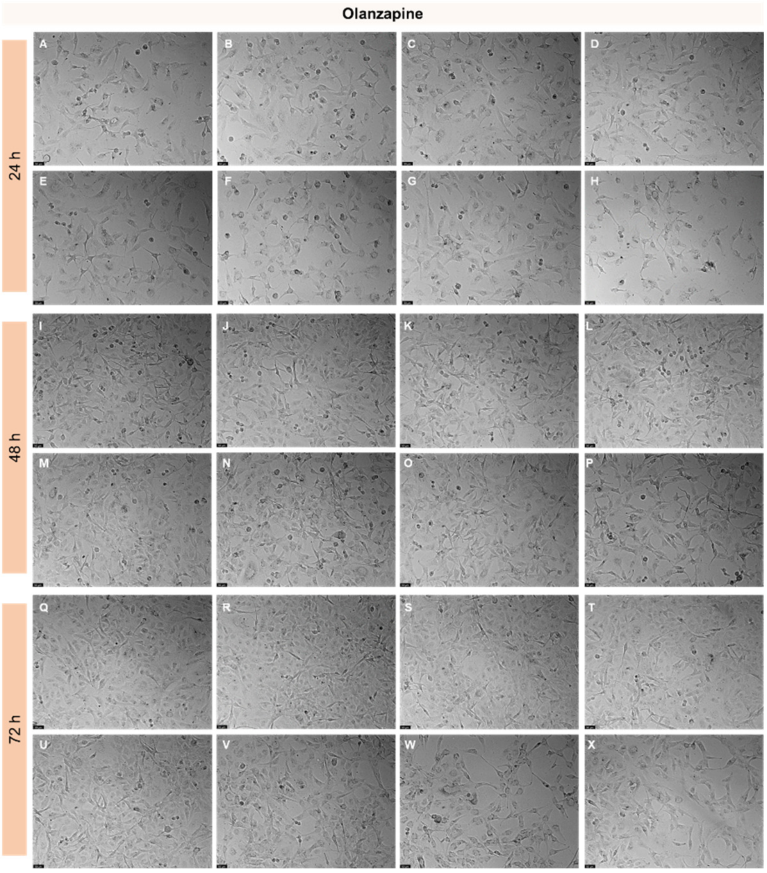
Olanzapine
24 h
48 h
72 h
A
B
C
D
E
F
G
H
I
J
K
L
M
N
O
P
Q
R
S
T
U
V
W
X

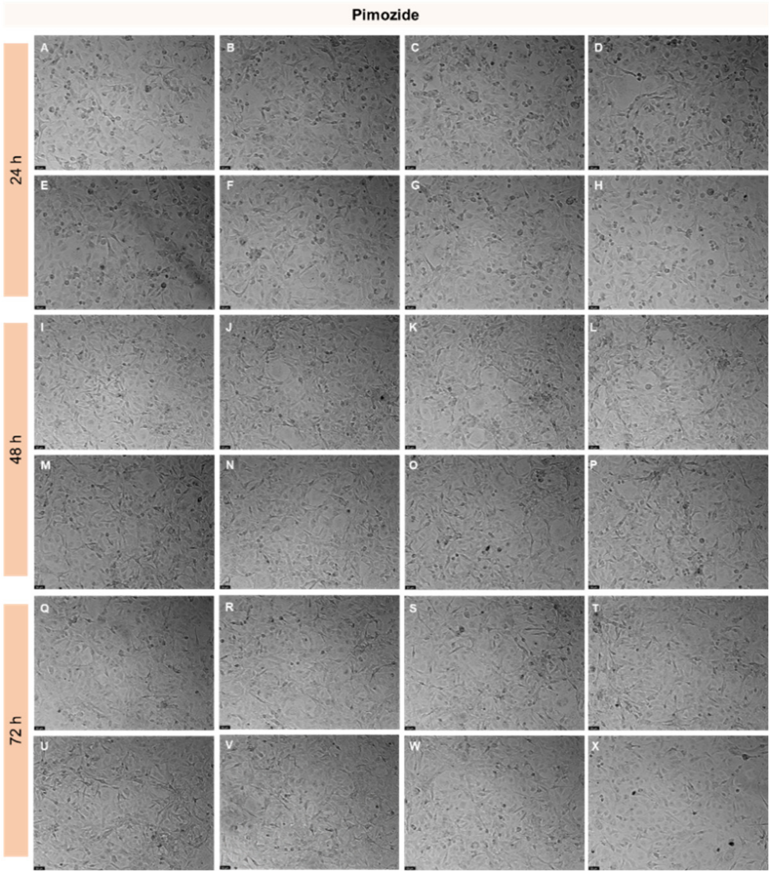

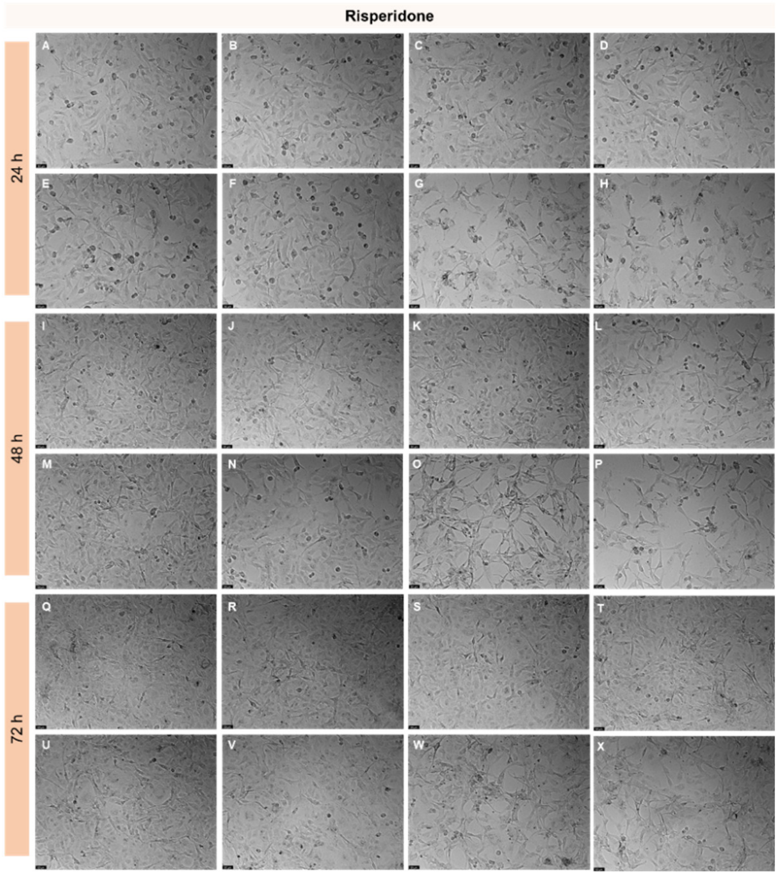

Figure S2. Microscopic visualization of the effects of CLZ, DOM, DRO, OLZ, PIM and RIS on the morphology of A172 cells over 24, 48 and 72 hours. Cells were treated with (**A,I,Q**) 0.1% DMSO (control), (**B,J,R**) 0.01

µM, (**C,K,S**) 0.1 µM, (**D,L,T**) 1 µM, (**E,M,U**) 10 µM, (**F,N,V**) 25 µM, (**G,O,W**) 50 µM and (**H,P,X**) 100 µM of CLZ, DOM, DRO, OLZ, PIM and RIS. Representative images were obtained with a high contrast (10×) bright field objective (LionHeart FX Automated Microscope) from three independent experiments. Scale bar: 50 µm.

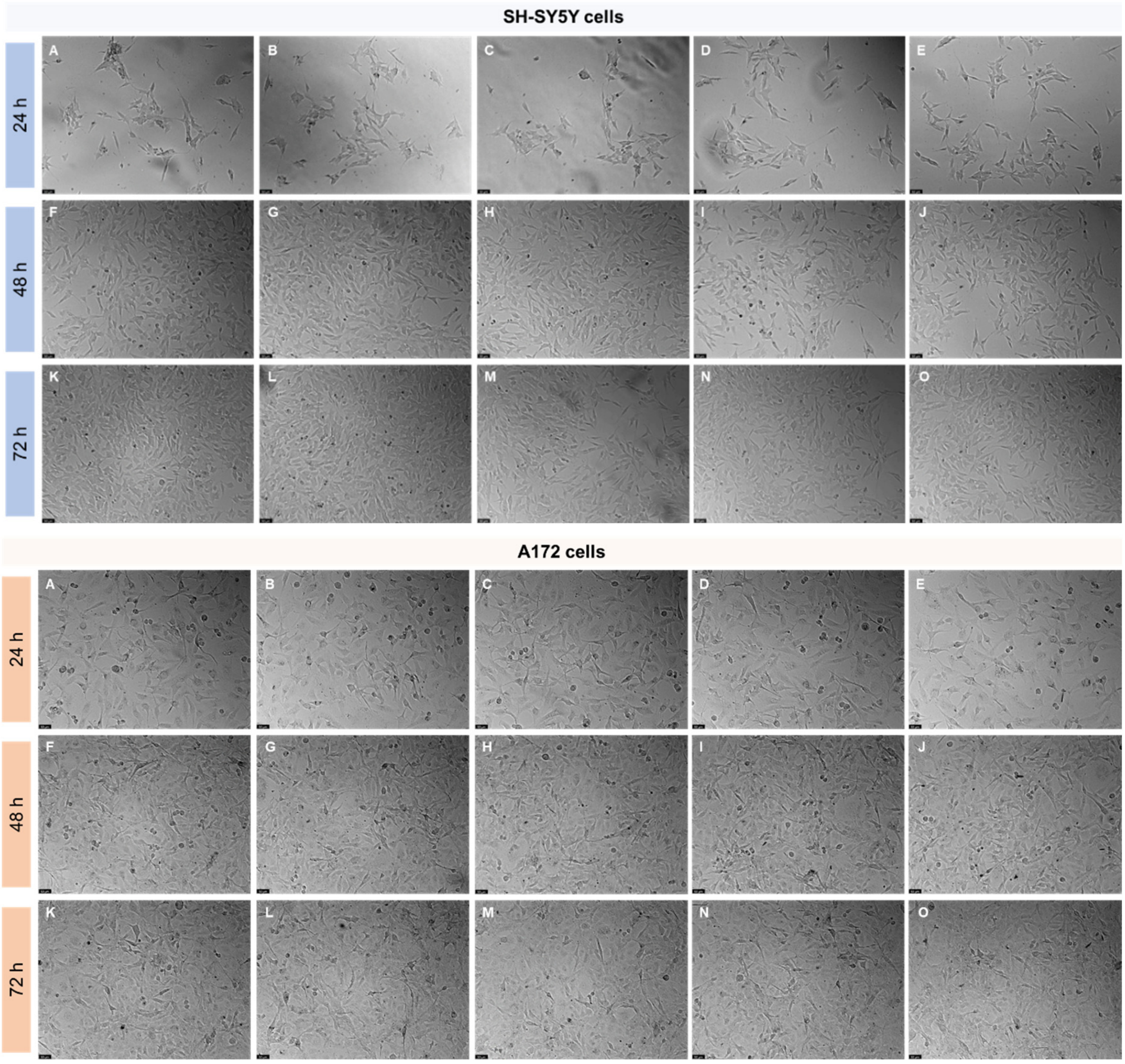

Figure S3. Microscopic visualization of the effects of Tyrosine on the morphology of SH-SY5Y and A172 cells over 24, 48 and 72 hours. Cells were treated with (**A,F,K**) 1% Water (control), (**B,G,L**) 25 µM, (**C,H,M**) 100 µM, (**D,I,N**) 250 µM and (**E,J,O**) 500 µM of Tyr. Representative images were obtained with a high contrast (10×) bright field objective (LionHeart FX Automated Microscope) from three independent experiments. Scale bar: 50 µM.

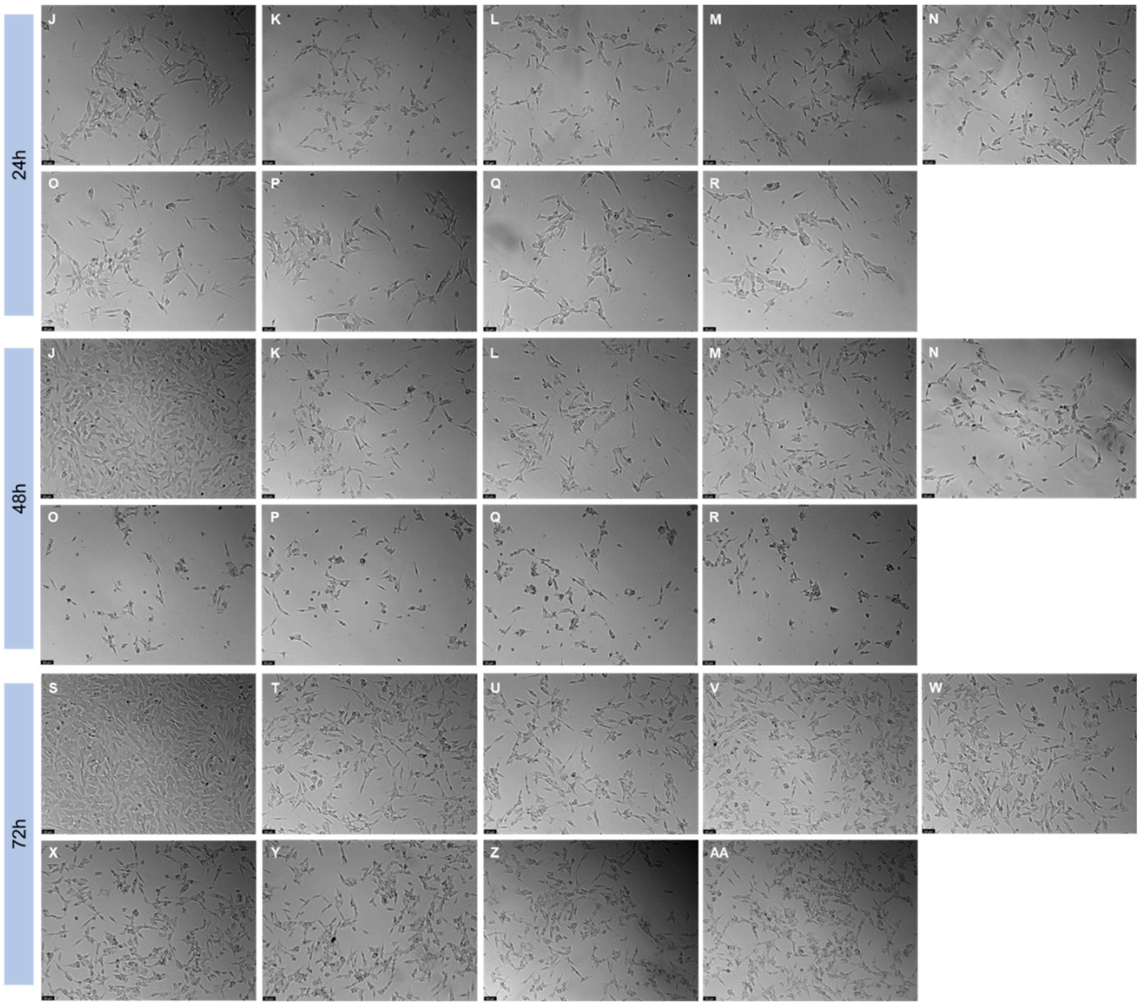

Figure S4. Microscopic visualization of the effects of CLZ combined with Tyr on the morphology of SH-SY5Y cells over 24, 48 and 72 hours. Cells were treated with (**A,J,S**) 0.1% DMSO (control), (**B,K,T**) CLZ 25 µM + Tyr 25 µM, (**C,L,U**) CLZ 25 µM + Tyr 100 µM, (**D,M,V**) CLZ 25 µM + Tyr 250 µM, (**E,N,W**) CLZ 25 µM + Tyr 500 µM, (**F,O,X**) CLZ 50 µM + Tyr 25 µM, (**G,P,Y**) CLZ 50 µM + Tyr 100 µM, (**H,Q,Z**) CLZ 50 µM + Tyr 250 µM and (**I,R,AA**) CLZ 50 µM + Tyr 500 µM. Representative images were obtained with a high contrast (10×) bright field objective (LionHeart FX Automated Microscope) from three independent experiments. Scale bar: 50 µm.

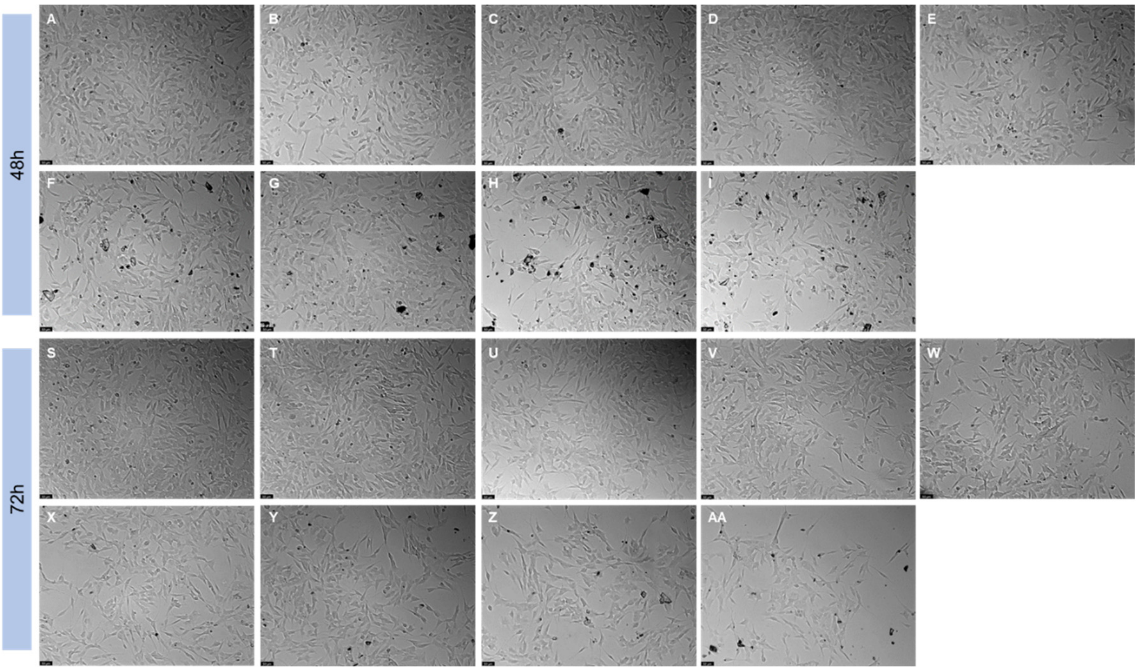

Figure S5. Microscopic visualization of the effects of DOM combined with Tyr on the morphology of SH-SY5Y cells over 48 and 72 hours. Cells were treated with (**A,J,S**) 0.1% DMSO (control), (**B,K,T**) DOM 10 µM + Tyr 25 µM, (**C,L,U**) DOM 10 µM + Tyr 100 µM, (**D,M,V**) DOM 10 µM + Tyr 250 µM, (**E,N,W**) DOM 10 µM + Tyr 500 µM, (**F,O,X**) DOM 25 µM + Tyr 25 µM, (**G,P,Y**) DOM 25 µM + Tyr 100 µM, (**H,Q,Z**) DOM 25 µM + Tyr 250 µM and (**I,R,AA**) DOM 25 µM + Tyr 500 µM. Representative images were obtained with a high contrast (10×) bright field objective (LionHeart FX Automated Microscope) from three independent experiments. Scale bar: 50 µm.

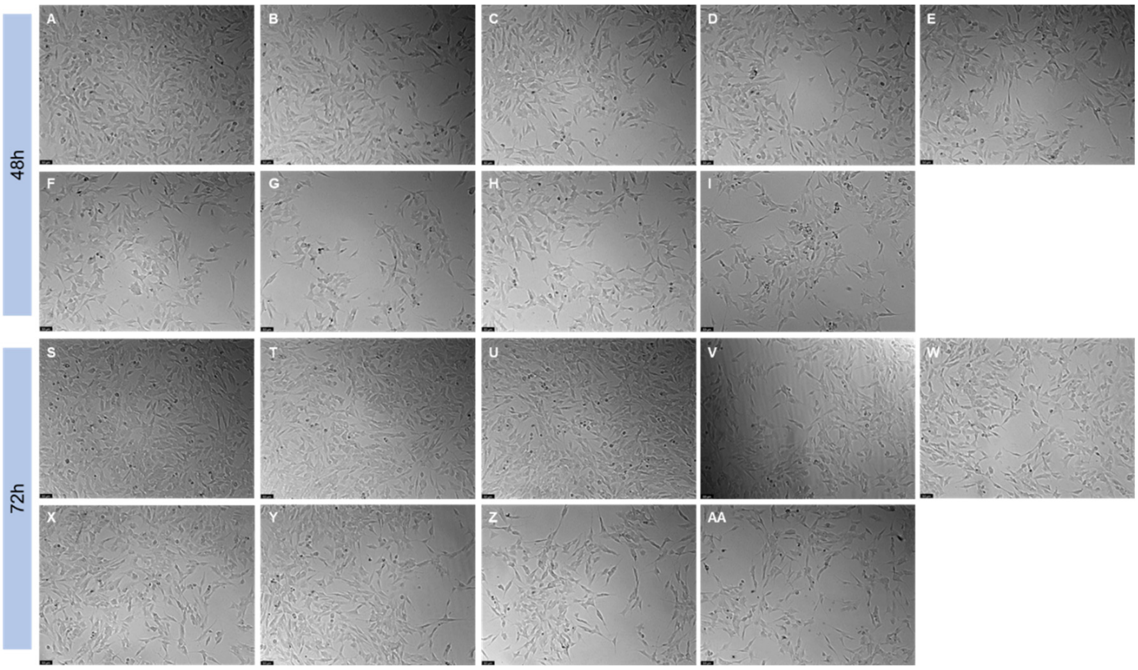

Figure S6. Microscopic visualization of the effects of PIM combined with Tyr on the morphology of SH-SY5Y cells over 48 and 72 hours. Cells were treated with (**A,J,S**) 0.1% DMSO (control), (**B,K,T**) PIM 1 µM + Tyr 25 µM, (**C,L,U**) PIM 1 µM + Tyr 100 µM, (**D,M,V**) PIM 1 µM + Tyr 250 µM, (**E,N,W**) PIM 1 µM + Tyr 500 µM, (**F,O,X**) PIM 10 µM + Tyr 25 µM, (**G,P,Y**) PIM 10 µM + Tyr 100 µM, (**H,Q,Z**) PIM 10 µM + Tyr 250 µM and (**I,R,AA**) PIM 10 µM + Tyr 500 µM. Representative images were obtained with a high contrast (10×) bright field objective (LionHeart FX Automated Microscope) from three independent experiments. Scale bar: 50 µm.

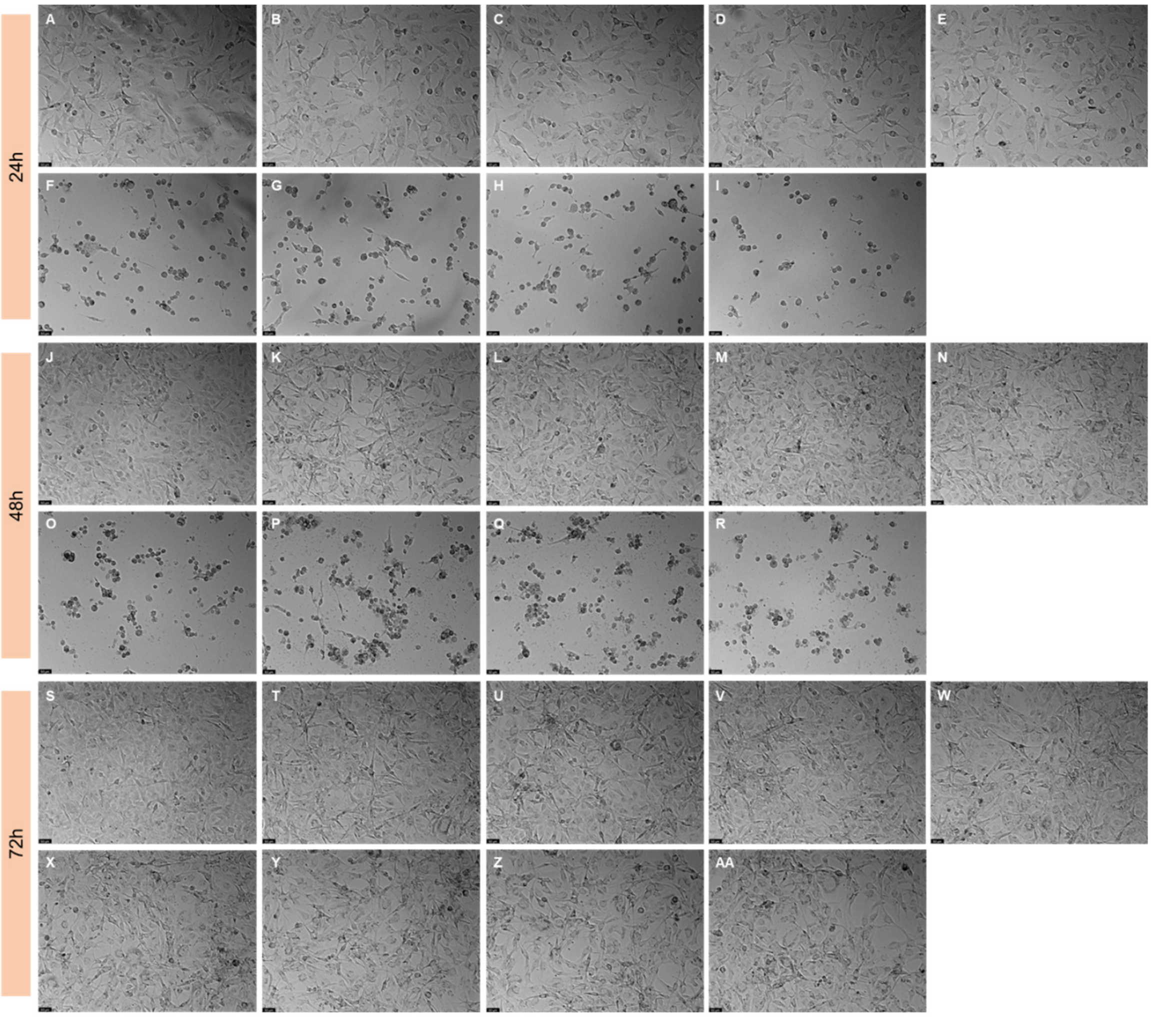

Figure S7. Microscopic visualization of the effects of CLZ combined with Tyr on the morphology of A172 cells over 24, 48 and 72 hours. Cells were treated with (**A,J,S**) 0.1% DMSO (control), (**B,K,T**) CLZ 25 µM + Tyr 25 µM, (**C,L,U**) CLZ 25 µM + Tyr 100 µM, (**D,M,V**) CLZ 25 µM + Tyr 250 µM, (**E,N,W**) CLZ 25 µM + Tyr 500 µM, (**F,O,X**) CLZ 50 µM + Tyr 25 µM, (**G,P,Y**) CLZ 50 µM + Tyr 100 µM, (**H,Q,Z**) CLZ 50 µM + Tyr 250 µM and (**I,R,AA**) CLZ 50 µM + Tyr 500 µM. Representative images were obtained with a high contrast (10×) bright field objective (LionHeart FX Automated Microscope) from three independent experiments. Scale bar: 50 µm.

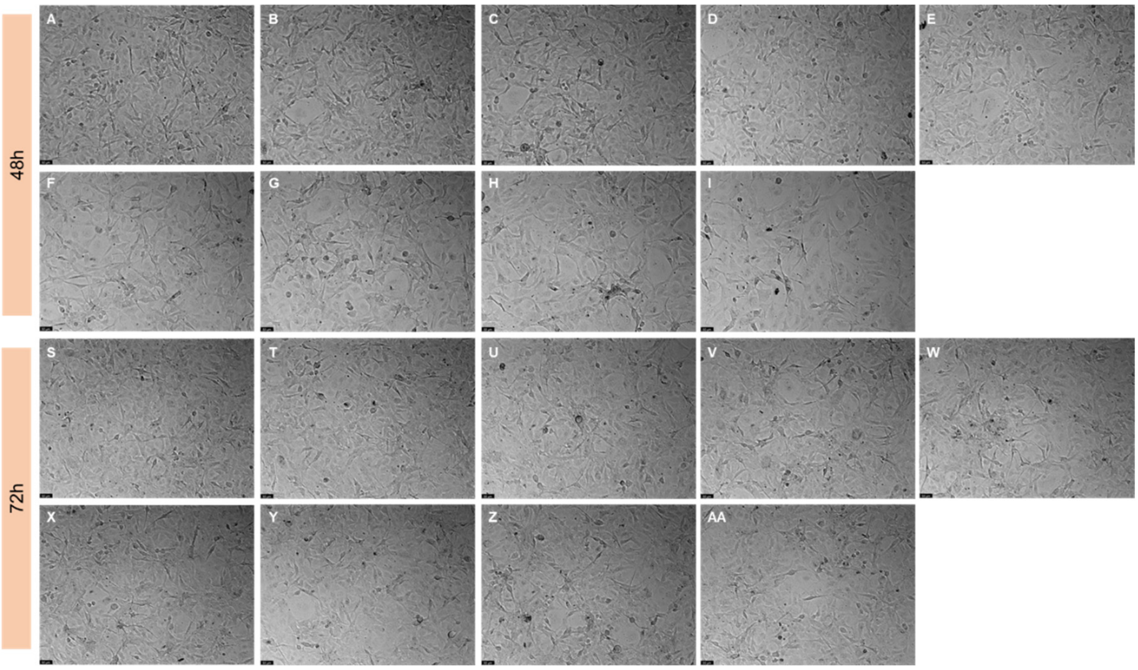

Figure S8. Microscopic visualization of the effects of RIS combined with Tyr on the morphology of A172 cells over 48 and 72 hours. Cells were treated with (**A,J,S**) 0.1% DMSO (control), (**B,K,T**) RIS 50 µM + Tyr 25 µM, (**C,L,U**) RIS 50 µM + Tyr 100 µM, (**D,M,V**) RIS 50 µM + Tyr 250 µM, (**E,N,W**) RIS 50 µM + Tyr 500 µM, (**F,O,X)** RIS 100 µM + Tyr 25 µM, (**G,P,Y**) RIS 100 µM + Tyr 100 µM, (**H,Q,Z**) RIS 100 µM + Tyr 250 µM and (**I,R,AA**) RIS 100 µM + Tyr 500 µM. Representative images were obtained with a high contrast (10×) bright field objective (LionHeart FX Automated Microscope) from three independent experiments. Scale bar: 50 µm.

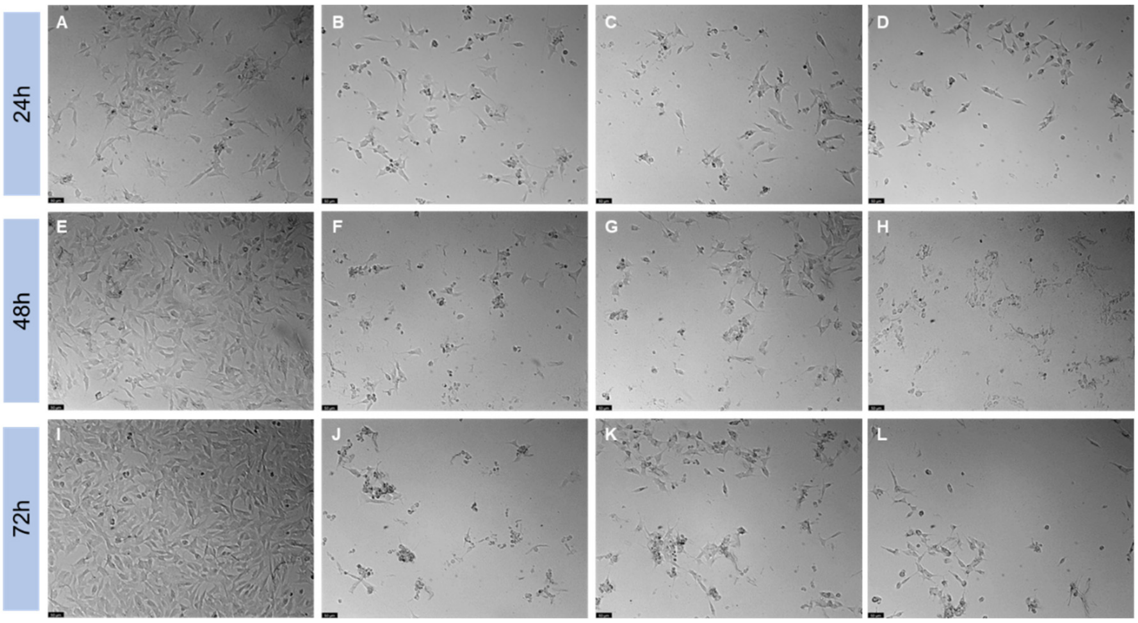

Figure S9. Microscopic visualization of the effects of CLZ combined with H_2O_2 on the morphology of SH-SY5Y cells over 24, 48 and 72 hours. Cells were treated with (**A,E,I**) 0.1% DMSO (control), (**B,F,J**) H_2O_2 132 µM, (**C,L,U**) CLZ 25 µM + H_2O_2 132 µM and (**D,M,V**) CLZ 50 µM + H_2O_2 132 µM. Representative images were obtained with a high contrast (10×) bright field objective (LionHeart FX Automated Microscope) from three independent experiments. Scale bar: 50 µm.

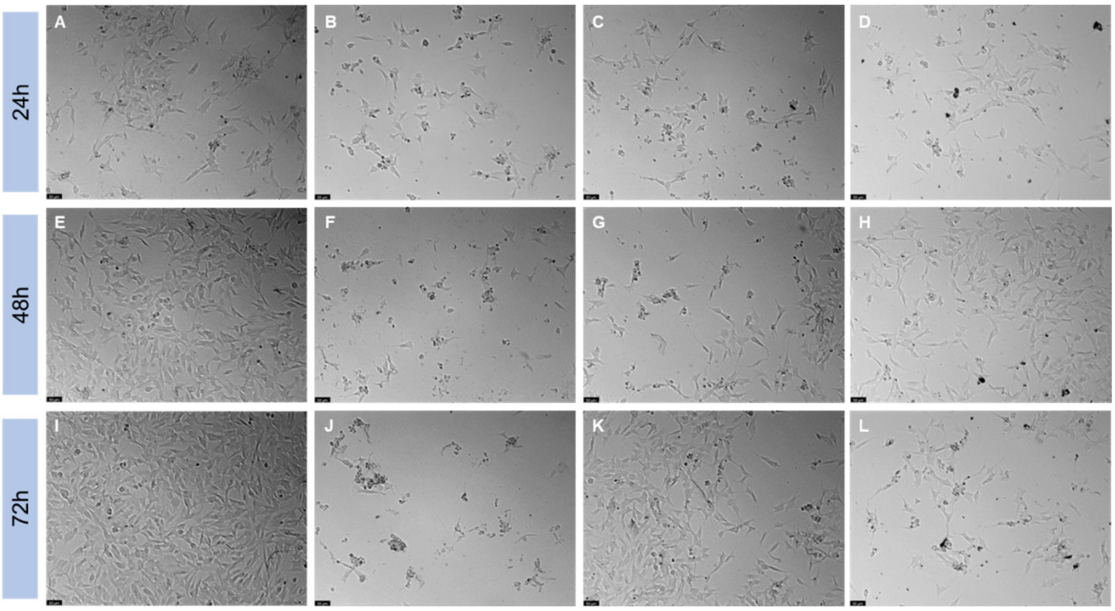

Figure S10. Microscopic visualization of the effects of DOM combined with H_2O_2 on the morphology of SH-SY5Y cells over 24, 48 and 72 hours. Cells were treated with (**A,E,I**) 0.1% DMSO (control), (**B,F,J**) H_2O_2 132 µM, (**C,L,U**) DOM 10 µM + H_2O_2 132 µM and (**D,M,V**) DOM 25 µM + H_2O_2 132 µM. Representative images were obtained with a high contrast (10×) bright field objective (LionHeart FX Automated Microscope) from three independent experiments. Scale bar: 50 µm.

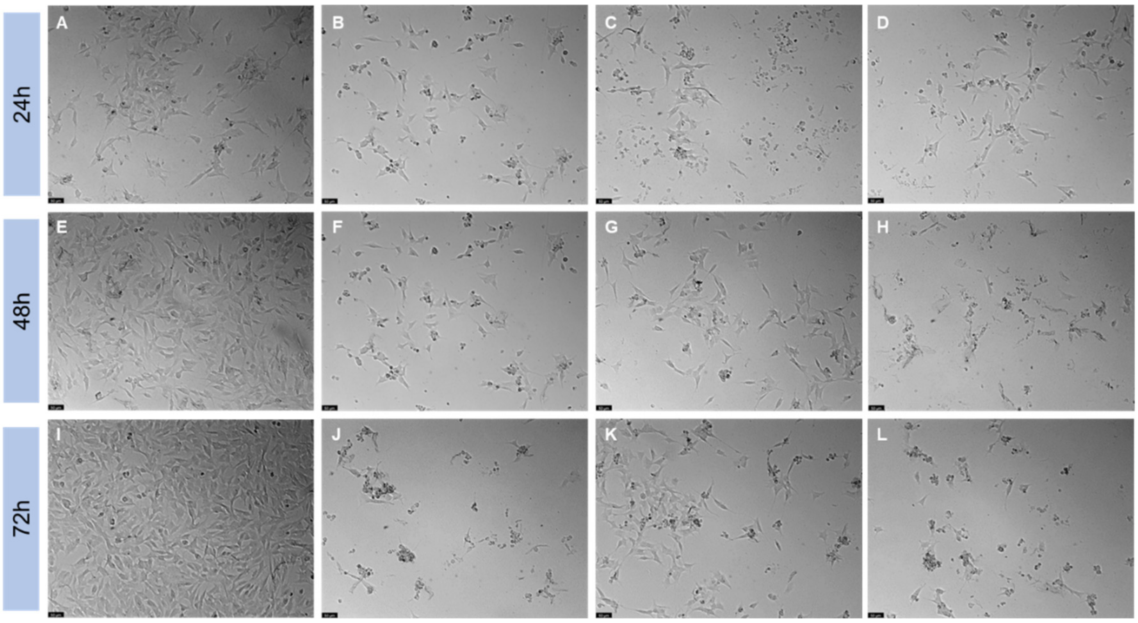

Figure S11. Microscopic visualization of the effects of PIM combined with H_2O_2 on the morphology of SH-SY5Y cells over 24, 48 and 72 hours. Cells were treated with (**A,E,I**) 0.1% DMSO (control), (**B,F,J**) H_2O_2 132 µM, (**C,L,U**) PIM 1 µM + H_2O_2 132 µM and (**D,M,V**) PIM 10 µM + H_2O_2 132 µM. Representative images were obtained with a high contrast (10×) bright field objective (LionHeart FX Automated Microscope) from three independent experiments. Scale bar: 50 µm.

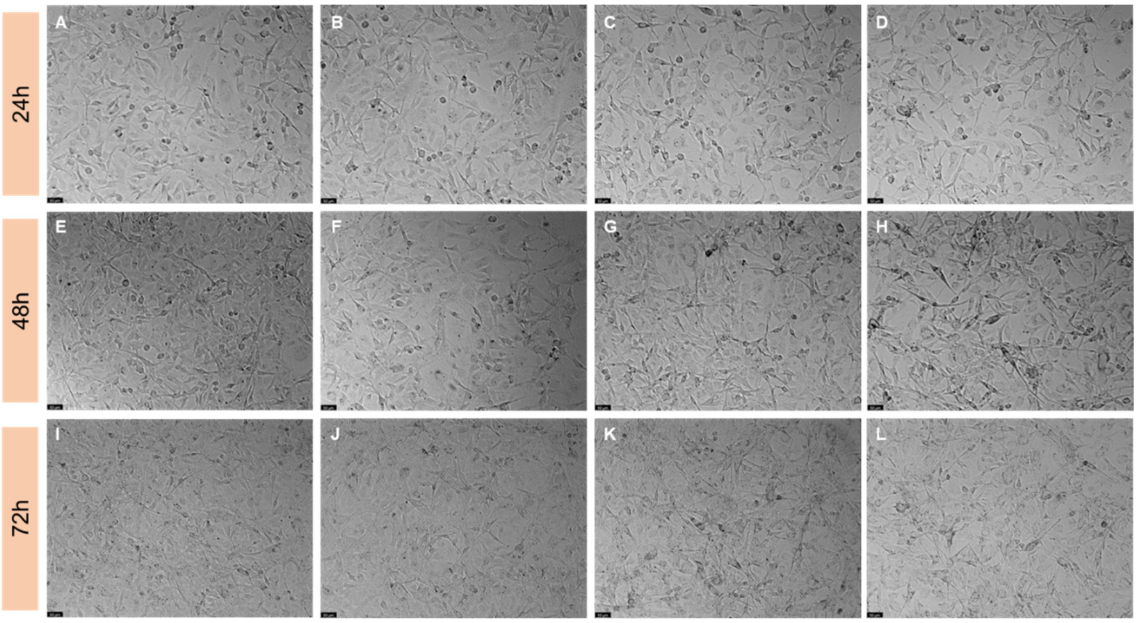

Figure S12. Microscopic visualization of the effects of CLZ combined with H_2O_2 on the morphology of A172 cells over 24, 48 and 72 hours. Cells were treated with (**A,E,I**) 0.1% DMSO (control), (**B,F,J**) H_2O_2 132 µM, (**C,L,U**) CLZ 25 µM + H_2O_2 132 µM and (**D,M,V**) CLZ 50 µM + H_2O_2 132 µM. Representative images were obtained with a high contrast (10×) bright field objective (LionHeart FX Automated Microscope) from three independent experiments. Scale bar: 50 µm.

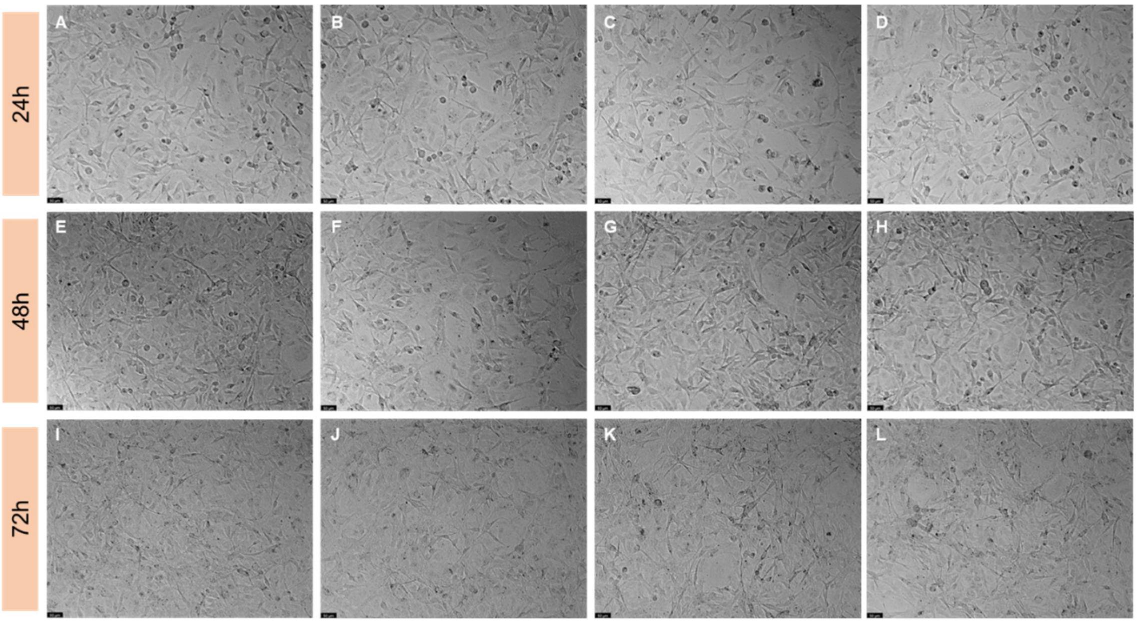

Figure S13. Microscopic visualization of the effects of RIS combined with H_2O_2 on the morphology of A172 cells over 24, 48 and 72 hours. Cells were treated with (**A,E,I**) 0.1% DMSO (control), (**B,F,J**) H_2O_2 132 µM, (**C,G,K**) RIS 50 µM+ H_2O_2 132 µM and (**D,H,L**) RIS 100 µM + H_2O_2 132 µM. Representative images were obtained with a high contrast (10×) bright field objective (LionHeart FX Automated Microscope) from three independent experiments. Scale bar: 50 µm.

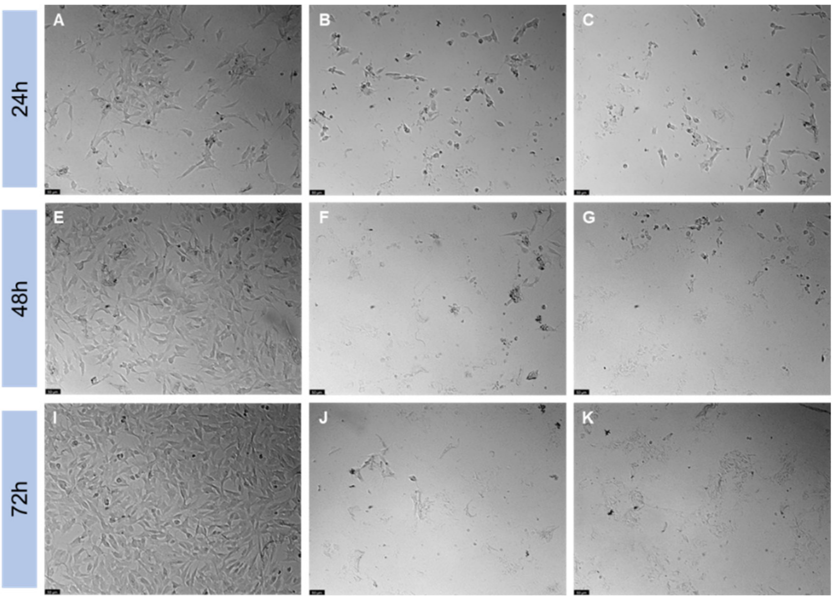

Figure S14. Microscopic visualization of the effects of CLZ combined with H_2O_2 and with Tyr on the morphology of A172 cells over 24, 48 and 72 hours. Cells were treated with (**A,D,G**) 0.1% DMSO (control), (**B,E,H**) CLZ 25 µM + H_2O_2 132 µM + Tyr 500 µM and (**C,F,I**) CLZ 50 µM + H_2O_2 132 µM + Tyr 500 µM. Representative images were obtained with a high contrast (10×) bright field objective (LionHeart FX Automated Microscope) from three independent experiments. Scale bar: 50 µm.

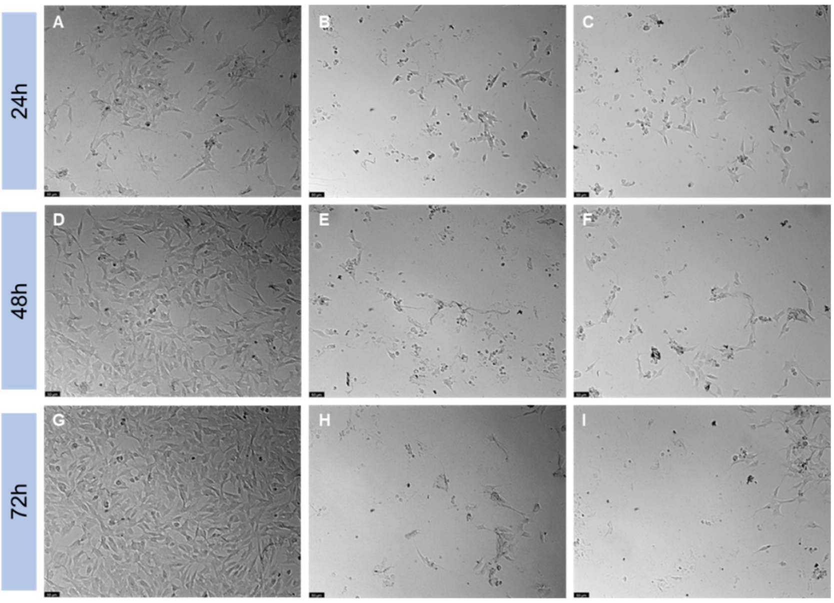

Figure S15. Microscopic visualization of the effects of DOM combined with H_2O_2 and with Tyr on the morphology of A172 cells over 24, 48 and 72 hours. Cells were treated with (**A,D,G**) 0.1% DMSO (control), (**B,E,H**) DOM 10 µM + H_2O_2 132 µM + Tyr 500 µM and (**C,F,I**) DOM 25 µM + H_2O_2 132 µM + Tyr 500 µM. Representative images were obtained with a high contrast (10×) bright field objective (LionHeart FX Automated Microscope) from three independent experiments. Scale bar: 50 µm.

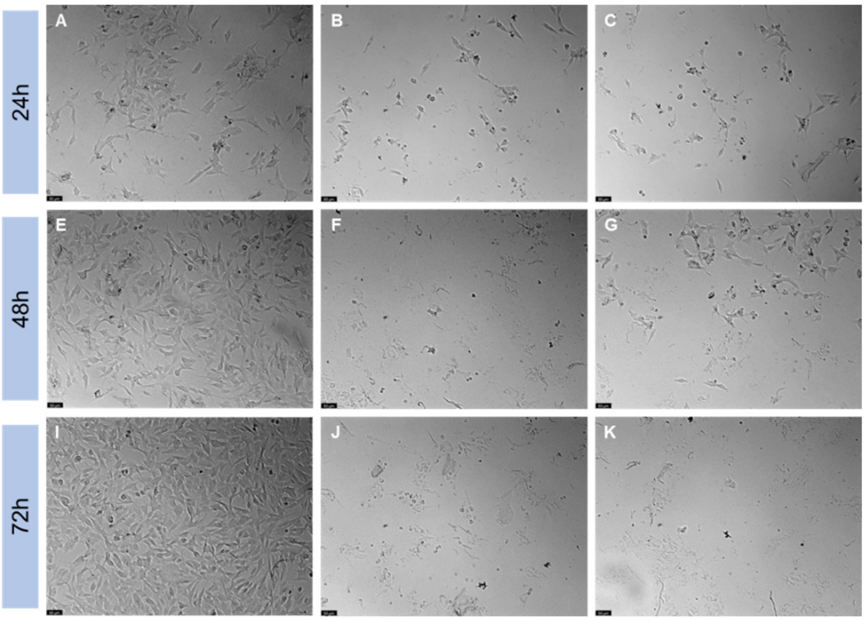

Figure S16. Microscopic visualization of the effects of PIM combined with H_2O_2 and with Tyr on the morphology of A172 cells over 24, 48 and 72 hours. Cells were treated with (**A,D,G**) 0.1% DMSO (control), (**B,E,H**) PIM 1 µM + H_2O_2 132 µM + Tyr 500 µM and (**C,F,I**) PIM 10 µM + H_2O_2 132 µM + Tyr 500 µM. Representative images were obtained with a high contrast (10×) bright field objective (LionHeart FX Automated Microscope) from three independent experiments. Scale bar: 50 µm.

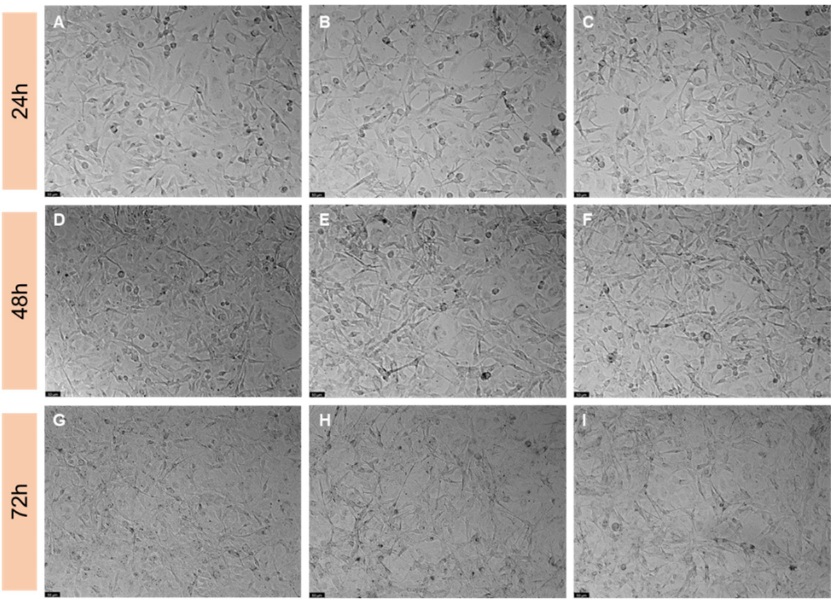

Figure S17. Microscopic visualization of the effects of CLZ combined with H_2O_2 and with Tyr on the morphology of A172 cells over 24, 48 and 72 hours. Cells were treated with (**A,D,G**) 0.1% DMSO (control), (**B,E,H**) CLZ 25 µM + H_2O_2 132 µM + Tyr 500 µM and (**C,F,I**) CLZ 50 µM + H_2O_2 132 µM + Tyr 500 µM. Representative images were obtained with a high contrast (10×) bright field objective (LionHeart FX Automated Microscope) from three independent experiments. Scale bar: 50 µm.

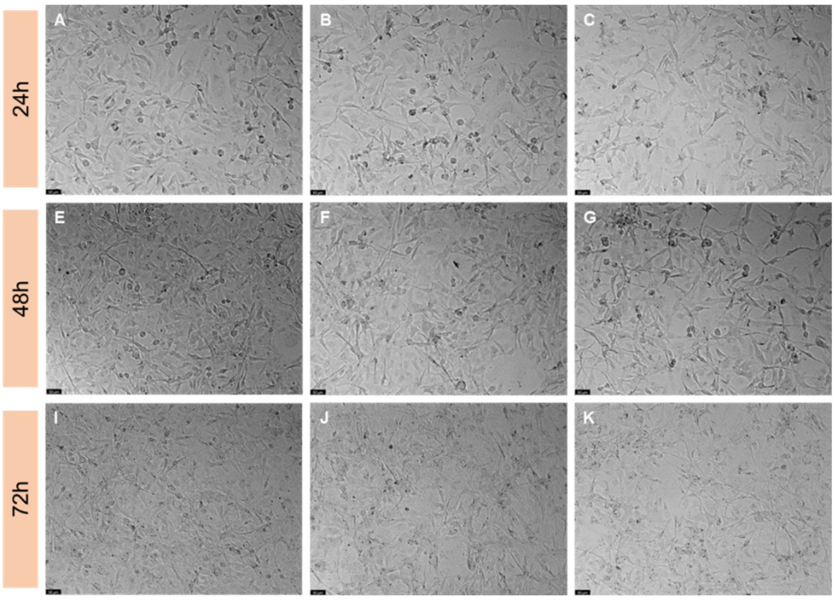

Figure S18. Microscopic visualization of the effects of RIS combined with H_2O_2 and with Tyr on the morphology of A172 cells over 24, 48 and 72 hours. Cells were treated with (**A,E,I**) 0.1% DMSO (control), (**B,E,H**) RIS 50 µM + H_2O_2 132 µM + Tyr 500 µM and (**C,F,I**) RIS 100 µM + H_2O_2 132 µM + Tyr 500 µM. Representative images were obtained with a high contrast (10×) bright field objective (LionHeart FX Automated Microscope) from three independent experiments. Scale bar: 50 µm.
